# Mental health trajectories after juridical divorce: Does personality matter?

**DOI:** 10.1111/jopy.12737

**Published:** 2022-06-22

**Authors:** Gert Martin Hald, Cathrine Lawaetz Wimmelmann, Camilla S. Øverup, Ana Cipric, Søren Sander, Jenna Marie Strizzi

**Affiliations:** ^1^ Department of Public Health University of Copenhagen Copenhagen Denmark

**Keywords:** anxiety, big five personality, depression, divorce, somatization, stress

## Abstract

**Introduction:**

This study investigated whether the Big Five personality dimensions were associated with mental health trajectories and/or intervention effects of a digital divorce intervention from juridical divorce to 12 months following juridical divorce. The study utilized a randomized controlled trial study design (*N* = 676) and measured mental health outcomes (anxiety, depression, somatization, and stress) at study inclusion (i.e., at juridical divorce) and 3‐, 6‐, and 12 months after juridical divorce. Big Five personality dimensions were measured 1 month post study inclusion.

**Results:**

The study found that neuroticism is the personality dimension most predictive of post‐divorce mental health outcomes. Specifically, divorcees with higher neuroticism scores indicated worse mental health immediately following divorce, but their symptom levels decreased more rapidly over a 12 months period after juridical divorce compared with lower neuroticism divorcees. It is also notable that their mean scores for the mental health outcomes remained higher at all time points (3, 6, and 12 months post baseline), relative to those lower in neuroticism.

**Conclusion:**

Findings are discussed in light of divorce‐adjustment‐theory and the stress‐buffering model.

## 1 INTRODUCTION

In Denmark, almost half of all marriages end in divorce, reflecting the divorce rates in most industrialized countries (Statistics Denmark ‐ Divorce, 2020). This is alarming as divorce has been listed among the most stressful life events in modern societies (Cohen et al., 2016; Dohrenwend et al., 1978; Freeman et al., 2008; Holmes & Rahe, 1967) and is generally associated with elevated levels of mental distress, depressive symptoms, anxiety symptoms, somatization symptoms, anger, stress, hopelessness, and reduced overall mental and physical health (Amato, 2010; Hald, Cipric, et al., 2020; Malgaroli et al., 2017; Sander, Strizzi, et al., 2020; Strizzi et al., 2021).

Nonetheless, in accordance with divorce‐stress‐adjustment theory (Amato, 2000), a growing body of research has found great variability in the psychological adjustment to divorce (Hetherington, 2003; Malgaroli et al., 2017; Mancini et al., 2011; Perrig‐Chiello et al., 2015). Divorce‐stress‐adjustment theory (Amato, 2000) describes a wide range of risk and protective factors that may increase or decrease the risk of mental distress and affect mental health following divorce (Hald, Cipric, et al., 2020; Sander, Hald, et al., 2020), with some divorcees adjusting rapidly to their new life situation, and others enduring more prolonged or profound symptoms of distress and reduced mental health (Hetherington, 2003; Perrig‐Chiello et al., 2015). According to divorce‐stress‐adjustment theory, the severity of the psychological reaction to divorce and the length of the adaptation period have been suggested to depend on individual dispositions and intra‐ and interpersonal resources (Amato, 2010; Perrig‐Chiello et al., 2015), such as good coping and social skills, and strong support from friends and family. In this regard, not much is known about personality as a potential determinant of individuals' trajectories of mental distress or psychological adjustment after divorce over time.

Personality dimensions, or traits, describe individual differences in stable behavioral dispositions, including ways of feeling, thinking, and acting, and is an important determinant of how we react in and cope with different situations (DeYoung, 2015; Digman, 1990; Habashi et al., 2016; Roberts et al., 2014). During the last few decades, there has been a growing interest in investigating the influence of personality on health and psychological well‐being after major life events (Robins, 1995; Spinhoven, Elzinga, et al., 2011; Spinhoven, Roelofs, et al., 2011). This research reflects the stress‐buffering model (Wheaton, 1985), which emphasizes how risk factors and protective factors may modify the effects of stressful situations on psychological well‐being, including the development of symptoms of affective disorders. Small moderating effects of personality has been found regarding symptoms of anxiety and depression after major life events like spousal loss (Spinhoven, Roelofs, et al., 2011), with extraversion and conscientiousness driving these effects as protective factors (Pai & Carr, 2010), though another study found no lasting moderating effects of personality on depressive symptoms (Mitchell et al., 2021). More research has been done regarding the moderating effects of personality after major life events in the area of life satisfaction (LS). Personality has been associated with poorer adjustment to retirement (Hansson et al., 2020; higher neuroticism linked with lower LS), disability (Boyce & Wood, 2011; higher agreeableness predicted better and faster adaptation), and unemployment (Boyce et al., 2010; Hahn et al., 2015; higher conscientiousness and lower extraversion associated with lower drops in LS). However, other studies found that personality did not moderate reactions to life events such as marriage and widowhood (Anusic et al., 2014; Yap et al., 2012).

The most widely used model of personality structure in recent decades has been the Big Five model (Digman, 1990), which describes five broad personality dimensions: neuroticism, extraversion, openness, agreeableness, and conscientiousness. Among these, the most studied personality dimension in health research is neuroticism and there is solid evidence of associations between this trait and a wide range of negative psychological factors, including depression, anxiety, somatization, stress, and lower satisfaction with life (e.g., Lahey, 2009; Wimmelmann et al., 2020). Neuroticism has also been called “negative affectivity” (Watson & Clark, 1984), because individuals high on neuroticism tend to experience more negative affect, be emotionally unstable, and have more intense reactions to negative situations compared with individuals low on neuroticism (Eysenck & Eysenck, 1975). It is generally assumed that individuals high on neuroticism are more susceptible to negative experiences than individuals low on neuroticism because of this enhanced emotional reactivity. Furthermore, individuals high on neuroticism also tend to cope more inappropriately with negative emotions and stressful events (Wrosch et al., 2003). Therefore, it may be that neuroticism moderates psychological adjustment to post‐divorce life, such that divorcees higher in neuroticism have worse mental health adjustment after divorce as compared with divorcees with lower neuroticism. While the causal mechanism linking neuroticism with poor mental health is unclear, three hypotheses have been proposed by Lahey (2009). The first suggests that there may be similar genetic underpinnings influencing both neuroticism and mental health concerns. The second posits that people high on neuroticism increase their experiences of negative life events through their behavior and interactions, including experiencing more conflicted and unstable relationships, which can limit their social support network. Additionally, they perceive negative events as more stressful. The third regards emotional reactivity, in that people high on neuroticism experience more frequent and intense negative emotions in response to stressful life events. The final hypothesis was tested by Mitchell et al. (2021) in terms of whether neuroticism moderated the association between stressful life events (such as divorce) and depressive symptoms over a 2‐year period. Their findings suggest that the emotional reactivity to stressful life events and corresponding adversely affected mental health are time limited, in that the effects are stronger in the first 3‐months following the stressful life event and dissipate over time. In this vein, a recent meta‐analysis revealed that high neuroticism is associated with more intense emotion (i.e., higher scores of symptom severity), however, their findings did not support a link between neuroticism and emotional variability (Kalokerinos et al., 2020).

For various reasons, other Big Five personality dimensions may also be relevant for levels of mental health following divorce: Individuals high on extraversion tend to experience positive emotions and have good social support, which has been associated with less mental distress after divorce (Kołodziej‐Zaleska & Przybyła‐Basista, 2016). Openness describes individuals who tend to be superior in adapting to new situations (DeYoung, 2015; Digman, 1990) and people higher in openness may therefore display faster psychological adjustment after a divorce. Individuals high on agreeableness are empathetic, tactful, trusting, and cooperative (Digman, 1990; Habashi et al., 2016), and they may therefore provide better conditions for a “low conflict” divorce. This could in turn reduce the risk of poorer mental health following divorce, as increased conflict levels have consistently been found to be associated with worse mental health following divorce (Hald, Strizzi, et al., 2020). Furthermore, agreeableness has been associated with the use of coping strategies that engage or protect social relationships, including support‐seeking (Lee‐Baggley et al., 2005), which has been suggested as important protective factors by divorce‐stress‐adjustment theory (Amato, 2000). Finally, conscientiousness describes individuals who are structured, responsible, and goal‐directed (Digman, 1990; Roberts et al., 2014), and this dimension has been associated with the use of problem‐focused—as opposed to emotion‐focused—coping strategies in stressful life situations (Lee‐Baggley et al., 2005), which may extend to divorce also.

In the divorce literature more generally, specific personality traits—especially the trait of neuroticism—have consistently been associated with marital dissolution and marital dissatisfaction (Holland & Roisman, 2008; Malouff et al., 2010; O'Meara & South, 2019). However, few studies have investigated the influence of personality traits on trajectories of mental health over time after divorce. In a cross‐sectional study of 308 divorced individuals, Perrig‐Chiello et al. (2015) found that better psychological adjustment after a divorce—measured in terms of satisfaction with life, depression, health, mourning, and hopelessness—was associated with lower scores on neuroticism and higher scores on extraversion and openness. Also, Pudrovska and Carr (2008) found similar results using a prospective design; however, in this study, the described associations were significant only among women. Thus, in the literature, there are indications that individuals with higher emotional stability, a more social profile, and better adaptability and openness to new situations are better psychologically adjusted after a divorce.

However, the literature on the effects of personality on mental health after divorce is scarce and the predictive value of personality for the course of symptoms of depression, anxiety, somatization, and stress after divorce remains unclear. For instance, a question that remains unanswered is whether personality is of predictive value for symptom trajectories after divorce or whether personality is more important for the immediate emotional reaction to the divorce. To our knowledge, no study has investigated the effects of the Big Five personality traits for several symptoms of mental health measured at multiple time points after divorce. Additionally, it is unclear whether personality is associated with improved outcomes following participation in an intervention (i.e., whether certain personality dimensions are associated with better intervention effects). In this regard, research that has looked into the possible moderating effects of personality in intervention studies have found that those high on agreeableness, conscientiousness, and extraversion experienced improved intervention effects (e.g., face‐to‐face cognitive interventions among elderly populations; Cerino et al., 2020). Other studies have found that extraversion and openness moderate the effectiveness of interventions (Senf & Liau, 2013; Wellenzohn et al., 2018), such that there were stronger intervention effects for those higher in these characteristics. Conversely, yet other studies find no moderation effect of personality (Wang et al., 2017). Knowledge regarding whether personality is predictive of symptom trajectories after divorce may allow for early identification of individuals, who are at increased risk of experiencing a longer adjustment period following divorce. Moreover, knowing whether personality is associated with improved intervention outcomes could help to indicate whether certain individuals should be targeted for intervention.

The overall aim of the current study is to longitudinally investigate the effects of the Big Five personality traits on depression, anxiety, somatization, and stress over a 1‐year period after juridical divorce. We use longitudinal data from a randomized controlled trial intervention study on the effect of a digital divorce platform on divorces' mental health (see also Hald, Cipric, et al., 2020) to examine the following research questions (RQ):

RQ1: Is personality associated with symptoms of anxiety, depression, somatization, and stress over the course of the first 12 months after juridical divorce?

RQ 2: Are there personality‐specific trajectories for symptoms of anxiety, depression, somatization, and stress over the course of the first 12 months after juridical divorce?

RQ3: Are the positive intervention effects of the Cooperation after Divorce online divorce intervention stronger for certain personality characteristics in relation to symptoms of anxiety, depression, somatization, and stress?

## 2 METHOD

### 2.1 Participants

A total of 676 people participated in the study (*N*_intervention_ = 377; *N*_control_ = 299), of which 446 were women and 230 were men. Participants had been married on average for approximately 13 years, were experiencing their first divorce, and had on average two children. Female participants were slightly younger and had higher educational attainment and incomes than male participants (see Table 1).

**TABLE 1:** Participant sociodemographic information and baseline mental and physical health scores (*N* = 676, men *n* = 230, women *n* = 446)

Variable	Men (*n* = 230)	Women (*n* = 446)
Age, years, mean (*SD*)	46.70 (9.31)	45.34 (8.05)*
Education level, %		
Low education level	30.4	28.3***
Medium education level	39.6	27.1
High education level	30.0	44.6
Income, %		
Below the average national monthly salary	30.0	12.3***
Average	23.5	43.9
Above the average national monthly salary	46.5	44.6
Marriage length, mean (*SD*)	12.92 (7.97)	13.84 (8.25)
Number of times divorced, %		
One time	89.1	88.8
Two or more times	10.9	11.1
Number of children, *M* (*SD*)	1.92 (0.89)	1.93 (0.96)
Baseline anxiety scale, *M* (*SD*), min‐max 0–4	0.82 (0.77)	0.94 (0.80)*
Baseline depression scale, *M* (*SD*), min‐max 0–4	1.38 (0.94)	1.51 (0.94)*
Baseline somatization scale, *M* (*SD*), min‐max 0–4	0.61 (0.64)	0.87 (0.73)**
Baseline stress scale, *M* (*SD*), min‐max 0–40	18.55 (7.07)	20.06 (7.10)**
Neuroticism, *M* (*SD*), min‐max 1–45	20.22 (8.49)	23.13 (8.60)**
Extraversion, *M* (*SD*), min‐max 0–48	30.13 (7.17)	28.84 (7.36)
Openness, *M* (*SD*), min‐max 7–45	27.93 (6.34)	29.93 (6.34)*
Agreeableness, *M* (*SD*), min‐max 14–48	31.76 (5.88)	34.62 (5.10)**
Conscientiousness, *M* (*SD*), min‐max 13–48	32.85 (5.10)	33.96 (6.06)*

*Notes*: *p < .05, **p < .01, ***p < .001, for gender differences, Min = minimum score for the sample; max = maximum score for the sample.

Compared with all people who divorced in Denmark during the study period, using data obtained from Statistics Denmark, participants in our study differed in terms of gender (there were more female participants in the present study; *χ* ^2^ [1, *n* = 676] = 69.02, *p* < .001), educational level (participants had higher educational attainment; *χ* ^2^ [2, *n* = 676] = 1135.23, *p* < .001), income (participants had a higher incomes, *t*[675] = 2.50 *p* = .013), and number of previous divorces (had been divorced fewer times, *t*[675] = −5.91, *p* < .001). However, participants were representative in age and marriage duration (*p* > .05).

We did experience significant attrition (*N*_baseline_ = 676; *N*_3‐month_ = 370; *N*_6‐month_ = 333; *N*_12‐month_ = 275). To assess for possible attrition bias, participants who stayed in the study beyond baseline (i.e., responded to the 3‐month follow‐up questionnaire) were compared with those who only completed the baseline questionnaire on intervention group assignment, sociodemographic variables (gender, age, number of children), socioeconomic variables (educational level, income), divorce‐related variables (duration of previous marriage, number of divorced, divorce conflict), health outcomes (depression, anxiety, and somatization), and personality variables (agreeableness, neuroticism, extraversion, openness, conscientiousness). The results of multiple logistic regression analyses showed that those who dropped out were more likely to have a medium education level (relative to low educational level; AOR = 1.81, *p* = .019), to have higher income (AOR = 1.167, *p* = .027), to have lower anxiety (AOR = −0.606, *p* = .046), and to be less extraverted (AOR = 0.95, *p* = .004).

### 2.2 Procedure

The data come from the “Cooperation after Divorce” (CAD) study, a 12‐month online intervention randomized controlled trial (RCT). The CAD RCT intervention aimed to reduce well‐known adverse physical and mental health symptoms after divorce, including health‐related quality of life, perceived stress, anxiety, depression, hostility, somatization, parent reports of children's health‐related quality of life, sick days, and days of absence from work. The CAD study utilized an online digital divorce platform consisting of 17 theme‐specific learning modules (e.g., grief, children's reactions after divorce, and conflict management) that participants in the intervention group could access at any time over a 12 months period, after responding to baseline questions (please see Cipric et al., 2020; Hald, Cipric, et al., 2020; Øverup et al., 2020; Sander, Hald, et al., 2020). Recruitment of participants was carried out in collaboration with the Danish State Administration (DSA). Before 2019, Danish residents seeking to obtain a divorce filed an application with the DSA; in mutual agreement cases, a divorce decree was processed and received in approximately 2 to 3 weeks. For approximately 30% of divorces during the study period, a 6‐month separation period was initiated before the divorce decree was granted because one of the spouses disagreed with the divorce or its terms. Between January 2016 and January 2018, the DSA distributed, along with divorce decrees, study invitation letters that included study participation links. Participation in the study was initiated by interested divorcees by using the link in the invitation letter, creating an account on the CAD webpage, providing informed consent, and completing the initial survey. After this, participants were randomized into the intervention or control group. Follow‐up surveys, which included measures of anxiety, depression, somatization symptoms, and stress, were sent by email at 3, 6, and 12 months after completion of the initial survey (this was responded to on average within 5 days of legal divorce). All data were stored on a secure server and anonymized. This research was approved by the Danish Data Protection Agency and was exempt from further ethical evaluations following the rules and regulations as set forth by the Scientific Ethical Committees of Denmark. For detailed information regarding the CAD intervention and its effectiveness, please refer to Cipric et al. (2020), Hald, Cipric, et al. (2020), Øverup et al. (2020), and Sander, Hald, et al. (2020).

### 2.3 Materials and measures

#### 2.3.1 Sociodemographic variables

The following sociodemographic variables were included in the study. The response options for each are also indicated. (1) gender: 0 = male and 1 = female, (2) age at divorce (in years), (3) education level (the highest level of completed formal education): 0 = low level of education (e.g., primary school, high school, business high school, vocational education), 1 = medium level of education (e.g., medium‐cycle tertiary education, bachelor's degree), and 2 = high level of education (e.g., master's degree or higher). (4) Monthly income (9‐point scale, 0 = below 10,000 DKK (less than 1500 USD) to 8 = more than 80,000 DKK (more than 12,000 USD)). Monthly income was also divided into three categories, with 1 = “Below national average” (1–3; less than 30,000 DKK), 2 = “At the national average” (4–5; 30,001–50,000 DKK), and 3 = “Above the national average” (6–9; above 50,001 DKK) (1 DKK ~ 0.15 USD [during the study period]). These were categorized so that 0 = below the Danish national average monthly salary, 1 = at the Danish national average monthly salary, and 2 = above the Danish national average monthly salary.

#### 2.3.2 Marriage and divorce‐related variables

The variables and corresponding response options regarding the divorce and former marriage included in the study are as follows: (1) the number of previous divorces (number), (2) marriage duration (in years from marriage date to legal divorce date), and (3) the number of children (number).

#### 2.3.3 Outcome variables

The Danish version of the Symptom Checklist‐90–Revised (SCL‐90; Derogatis, 2009) was used to assess anxiety, depression, and somatization symptoms at the initial survey and the 3, 6, and 12‐month follow‐up time collections. The anxiety, depression, and somatization subscales included 10, 13, and 12 items, respectively. A 5‐point Likert‐type response scale was used, ranging from 0 = not at all to 4 = very much, with higher scores indicating higher levels of anxiety, depression, and somatization symptoms. High internal consistency of the subscales was observed at all time points (α = 0.86–0.95) of the present study. To assess stress, we used the Danish version of the 10‐item Perceived Stress Scale (PSS; Eskildsen et al., 2015). Participants responded on a five‐point Likert‐type response scale (0 = never, 4 = very often), with higher scores indicating higher levels of stress. Scores range from 0 to 40, with higher scores indicating higher perceived stress. The scale demonstrated good internal reliability at all measurement points in the current study (Cronbach's *α* = 0.90–0.92).

#### 2.3.4 Personality

The Danish language version of the NEO Five‐Factor Inventory (NEO‐FFI‐3) (Skovdahl‐Hansen et al., 2004) was used to assess the personality of the participants at the 1‐month post‐divorce time collection. The NEO‐FFI‐3 is a well‐validated questionnaire comprising 60 items and is a short version of the Revised NEO Personality Inventory (NEO PI‐R) (Costa & McCrae, 1992), which assesses the Big Five personality dimensions of neuroticism, extraversion, openness, agreeableness, and conscientiousness. All 60 items of the NEO‐FFI‐3 are answered on a five‐point Likert scale from 0 (strongly agree) to 4 (strongly disagree), corresponding to the American version. Thus, the total score range for the 12 items included for each personality dimension is 0–48. Cronbach's alphas for the five traits are reported to be 0.77–0.84 reflecting a high internal consistency of the Danish version of the test (Skovdahl‐Hansen et al., 2004). The internal consistency was also high for the present study (*α* = 0.73–0.88).

### 2.4 Data analyses

To evaluate the effect of the five personality dimensions on the outcomes, we employed linear mixed‐effect regression modeling with the lme4 package version 1.1–25 (Bates et al., 2015) for R version 3.5.3. We used likelihood ratio tests to assess the three models to determine whether there was an association between the personality dimension variables and the outcomes, and if so, the nature of these associations.

Model 1 assumed no effect of personality at all (personality variables were not included in analyses), model 2 assumed a simple additive effect of personality (time‐invariant; the main effect of the personality dimension), model 3 assumed personality dimension‐specific trajectories across time (personality dimension × time interaction), and model 4 assumed group by personality dimension‐specific trajectories across time (group × personality dimension × time interaction). As we conducted multiple comparisons tests, we adopted a Bonferroni correction, correcting for the numbers of test (within outcome). Thus, the corrected alpha value is 0.05/3 = 0.017 for the likelihood ratio tests.

**FIGURE 1:**
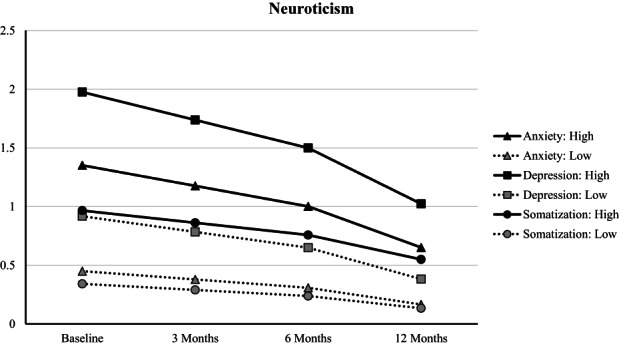
Depression, anxiety, and somatization in average scores over time by high and low neuroticism scores, as estimated by mixed linear effect models (model 3). All personality dimensions were entered simultaneously as predictors.

We controlled for RCT group placement (intervention and control group) over time, gender, educational level, and income in all models, and the effects reported reflect these adjusted models. For all analyses, the predictor variables were the personality dimension (neuroticism, extraversion, openness, agreeableness, and conscientiousness; entered simultaneously), time points, the interaction between personality and time, and the interaction between group assignment, personality, and time. Personality variables and time were treated as continuous, while intervention group placement was categorical. The personality variables were grandmean centered prior to analyses; interactions were plotted according to Aiken and West (1991), probing at ±1 *SD* from the mean (i.e., high and low values for the personality dimension; Figure 1). Individual differences in initial outcome variable score levels were accounted for with a random intercept; we also specified random slopes for time to allow for the fact that people may experience difference rates of change in the outcomes. We used the full information maximum likelihood (FIML) estimation approach in all linear mixed‐effect regression modeling to protect against informative missing patterns and to ensure the robustness of the longitudinal estimates (Little, 2013).

## 3 RESULTS

Table 2 presents zero‐order Pearson correlations between the predictor and covariates and outcome variables at each time point (for a full correlation matrix, please see the supplemental materials). Neuroticism was significantly and positively associated with all outcomes at all time points while extraversion and conscientiousness were significantly and negatively associated with all outcomes at all time points. Openness was generally not associated with the outcomes, while agreeableness was significantly and positively associated with the outcomes at 3 months only (see Table 2). All intervention group participants accessed the intervention platform (Mean number of modules accessed = 5.02, *SD* = 3.29, *Me* = 4).

**TABLE 2:** Correlations between personality dimensions and covariates and depression, anxiety, somatization, and stress

	Neuroticism	Extraversion	Openness	Agreeableness	Conscientiousness	Gender	Education level	Income
1. Neuroticism	–							
2. Extraversion	−0.519**	–						
3. Openness	−0.070	0.366**	–					
4. Agreeableness	−0.021	−0.010	0.144**	–				
5. Conscientiousness	−0.524**	0.312**	0.071	0.186**	–			
6. Anxiety (BL)	0.508**	−0.217**	0.035	0.047	−0.173**	0.071**	−0.159**	−0.167**
7. Depression (BL)	0.558**	−0.291**	−0.084*	0.064	−0.239**	0.064**	−0.182**	−0.185**
8. Somatization (BL)	0.445**	−0.206**	−0.005	0.061	−0.177**	0.172**	−0.164**	−0.202**
9. Stress (BL)	0.564**	−0.246**	−0.030	0.016	−0.276**	0.100**	−0.093*	−0.162**
10. Anxiety (3)	0.548**	−0.299**	0.091	0.116*	−0.211**	0.111*	−0.135**	−0.212**
11. Depression (3)	0.546**	−0.373**	−0.031	0.161**	−0.234**	0.118**	−0.138**	−0.229**
12. Somatization (3)	0.436**	−0.258**	0.009	0.116*	−0.184**	0.194**	−0.206**	−0.232**
13. Stress (3)	0.607**	−0.358**	−0.026	0.128*	−0.277**	0.189**	−0.057	−0.220**
14. Anxiety (6)	0.428**	−0.168**	0.158**	0.038	−0.140*	0.128**	−0.080	−0.136**
15. Depression (6)	0.502**	−0.255**	0.039	0.053	−0.234**	0.075	−0.121*	−0.198**
16. Somatization (6)	0.357**	−0.154**	0.035	0.098	−0.165**	0.191**	−0.108*	−0.179**
17. Stress (6)	0.543**	−0.222**	0.007	0.037	−0.263**	0.141*	−0.053	−0.147**
18. Anxiety (12)	0.417**	−0.244**	0.037	0.016	−0.213**	0.160**	−0.119*	−0.173**
19. Depression (12)	0.491**	−0.303**	−0.091	0.035	−0.247**	0.134**	−0.137**	−0.173**
20. Somatization (12)	0.308**	−0.177**	−0.038	0.043	−0.150*	0.164**	−0.157**	−0.215**
21. Stress (12)	0.500**	−0.245**	−0.090	−0.040	−0.307**	0.151*	−0.109	−0.123*
22. Gender	0.159**	−0.019	0.084*	0.245**	0.087*	–	0.067**	−0.243**
23. Educational level	−0.145**	0.140**	0.231**	−0.027	0.056		–	0.316**
24. Income	−0.284**	0.205**	0.045	−0.122**	0.160**			–

*Notes*: (BL) = at baseline, (3) = at 3 months, (6) = at 6 months, (12) = at 12 months.

* *p* < .05,

** *p* < .001.

Research question 1 and 2 concern the associations between personality and symptoms of anxiety, depression, somatization, and stress. The likelihood ratio tests indicated that, for three of the four outcomes, model 3, that is, a model with personality dimension‐specific trajectories across time (RQ2), had the best fit to data (Table 3). The likelihood tests did not support that a model that specified group by personality dimension‐specific trajectories across time fit the data better (RQ3). Examining the estimates for these model 3 results (in Table 4) suggested that there were consistent differences across time for neuroticism (for full results for models 1–4, please see the supplemental materials; the supplemental materials also contain univariate analyses in which each personality dimension is examined individually, as well as an examination of the correlations between individual rates of change for the outcomes). That is, individuals with higher neuroticism scores reported higher anxiety, depression, and somatization scores at baseline but sharper decreases in levels of anxiety, depression, and somatization symptoms over time (see Figure 1), relative to individuals with lower neuroticism scores. For stress, the results suggested that Model 2 (i.e., a main effects model) was the best fitting model; individuals with higher neuroticism scores reported higher stress scores.

**TABLE 3:** Results for the likelihood ratio tests comparing the three models tested for personality scores adjusted for gender, educational level, and income

Outcome	Model 1^a^ versus model 2^b^	Model 2^b^ versus model 3^c^	Model 3^c^ versus model 4^d^
Anxiety	*χ* ^2^(10) = 228.09, *p* < .001	*χ* ^2^(5) = 42.70, *p* < .001	*χ* ^2^(5) = 5.16, *p* = .397
Depression	*χ* ^2^(10) = 277.32, *p* < .001	*χ* ^2^(5) = 22.37, *p* < .001	*χ* ^2^(5) = 12.10 *p* = .033
Somatization	*χ* ^2^(10) = 128.67 *p* < .001	*χ* ^2^(5) =24.71, *p* < .001	*χ* ^2^(5) = 1.84, *p* = .870
Stress	*χ* ^2^(10) = 304.52, *p* < .001	*χ* ^2^(5) = 13.36, *p* = .020	*χ* ^2^(5) = 11.29, *p* = .046

*Notes*: A significant test comparing models 1 and 2 indicates that model 2 has the best fit to data; a significant test comparing models 2 and 3 indicates that model 3 has the best fit to data. Please note that we used a corrected alpha value is 0.05/3 = 0.017.

^a^ Model 1 assessed assumes no effect of personality.

^b^ Model 2 assessed for the main effect of personality.

^c^ Model 3 assessed for a personality by time interaction.

^d^ Model 4 assessed for a group by personality by time interaction.

**TABLE 4:** Results of linear mixed effect modeling (model 3) to assess the associations between all five personality dimensions and anxiety, depression, somatization symptoms, and stress scores, adjusted for gender, educational level, and income

Variable	Anxiety				Depression				Somatization				Stress			
	Estimate	*SE*	Cohen's *d*	*p*	Estimate	*SE*	Cohen's *d*	*p*	Estimate	*SE*	Cohen's *d*	*p*	Estimate	*SE*	Cohen's *d*	*p*
Intercept	**0.901**	**0.126**	**2.055**	**<.001**	**1.447**	**0.160**	**2.726**	**<.001**	**0.654**	**0.118**	**1.378**	**<.001**	**17.72**	**1.392**	**4.583**	**<.001**
Time	**−0.041**	**0.009**	**−0.092**	**<.001**	**−0.062**	**0.012**	**−0.117**	**<.001**	**−0.026**	**0.005**	**−0.055**	**<.001**	**−0.573**	**0.136**	**−0.148**	**<.001**
Group	−0.006	0.044	−0.014	.894	−0.003	0.056	−0.006	.954	0.030	0.044	0.063	.504	−0.035	0.426	−0.009	.934
Neuroticism	**0.052**	**0.005**	**0.119**	**<.001**	**0.061**	**0.006**	**0.115**	**<.001**	**0.036**	**0.005**	**0.076**	**<.001**	**0.463**	**0.042**	**0.120**	**<.001**
Extraversion	0.007	0.005	0.017	.146	0.005	0.006	0.009	.426	0.004	0.005	0.009	.381	0.085	0.045	0.022	.057
Openness	0.003	0.005	0.006	.598	−0.009	0.006	−0.017	.168	0.000	0.005	0.000	.972	−0.083	0.046	−0.022	.069
Agreeableness	−0.000	0.005	−0.000	.950	0.005	0.007	0.010	.444	0.001	0.005	0.002	.858	0.014	0.047	0.004	.767
Conscientiousness	**0.016**	**0.006**	**0.036**	**.006**	0.009	0.007	0.017	.216	0.008	0.006	0.018	.146	0.016	0.051	0.004	.752
Group × Neuroticism	−0.004	0.006	−0.010	.480	0.001	0.008	0.003	.855	−0.002	0.006	−0.004	.783	−0.014	0.058	−0.004	.806
Group × Extraversion	−0.013	0.007	−0.029	.074	−0.014	0.009	−0.026	.125	−0.006	0.007	−0.013	.389	−0.094	0.066	−0.024	.156
Group × Openness	**0.015**	**0.007**	**0.033**	**.041**	0.011	0.009	0.020	.230	0.003	0.007	0.007	.649	0.132	0.067	0.034	.050
Group × Agreeableness	0.010	0.008	0.022	.206	**0.019**	**0.010**	**0.037**	**.044**	0.009	0.008	0.020	.232	0.078	0.072	0.020	.280
Group × Conscientiousness	−0.003	0.008	−0.008	.679	−0.004	0.010	−0.007	.733	−0.009	0.008	−0.019	.289	−0.056	0.078	−0.015	.467
Time × Neuroticism	**−0.002**	**0.000**	**−0.006**	**<.001**	**−0.002**	**0.001**	**−0.004**	**<.001**	**−0.001**	**0.000**	**−0.003**	**<.001**				
Time × Extraversion	−0.001	0.000	−0.002	.075	−0.001	0.001	−0.002	.162	−0.000	0.000	−0.001	.299				
Time × Openness	0.000	0.000	0.000	.712	0.001	0.001	0.001	.236	−0.000	0.000	−0.000	.955				
Time × Agreeableness	−0.001	0.000	−0.001	.211	**−0.001**	**0.001**	**−0.003**	**.033**	−0.000	0.000	−0.001	.558				
Time × Conscientiousness	−0.001	0.001	−0.002	.094	−0.000	0.001	−0.001	.538	−0.000	0.000	−0.001	.524				
Time × Group	**0.031**	**0.005**	**0.071**	**<.001**	**0.050**	**0.007**	**0.093**	**<.001**	**0.023**	**0.005**	**0.049**	**<.001**	**0.440**	**0.054**	**0.114**	**<.001**
Gender	−0.012	0.047	−0.027	.803	0.011	0.059	0.020	.855	**0.140**	**0.048**	**0.296**	**.004**	0.316	0.440	0.082	.473
Education	−0.006	0.012	−0.014	.622	−0.014	0.015	−0.026	.372	−0.015	0.013	−0.032	.226	0.029	0.115	0.007	.803
Income	−0.010	0.013	−0.022	.475	−0.001	0.017	−0.002	.947	−0.019	0.014	−0.039	.170	0.021	0.125	0.005	.866

*Notes*: Bold indicates significance. For stress, we display the results of the main effects model. Full results for all analyses can be found in the supplemental materials.

Besides the finding for neuroticism, there were few consistent findings. There was a main effect of conscientiousness for anxiety, such that those with higher levels of conscientiousness reported higher levels of anxiety. There was a interactive effect between group assignment and openness, such that those in the control group with higher openness scores reported higher anxiety scores relative to those with lower openness scores (please see Figure S1, in the supplemental materials). Moreover, there was an interactive effect between group assignment and agreeableness, such that those in the control group with higher agreeableness scores reported higher depression scores relative to those with lower agreeableness scores (please see Figure S1, in the supplemental materials). And there was an interaction between time and agreeableness, such that individuals with higher agreeableness scores demonstrated sharper decreases in depression over time (see Figure S2), relative to individuals with agreeableness scores. Besides neuroticism, no personality dimensions were significant for somatization or stress (see Table 4 as well as supplemental materials for full results tables).

## 4 DISCUSSION

Overall, the results indicate that neuroticism is the personality dimension that is most predictive of post‐divorce adjustment in terms of anxiety, depression, and somatization symptoms. Those with higher neuroticism scores indicate higher levels of these outcome symptoms immediately following divorce relative to those with low neuroticism scores; however, their symptom levels decreased more rapidly over the next 12‐months, particularly from baseline to 3‐months, implying greater symptom improvements as compared with those lower in neuroticism. It should be noted that though their symptoms decreased more rapidly over the 12 months post‐divorce, the mean scores for depression, anxiety, somatization, and stress remained higher for those with higher neuroticism across time (3, 6, and 12 months post baseline), relative to those lower in neuroticism.

The findings of the current study are in line with past research, which has found that neuroticism is the primary personality factor associated with poor mental health, both in general (Lahey, 2009; Wimmelmann et al., 2020) and post‐divorce (Perrig‐Chiello et al., 2015; Pudrovska & Carr, 2008). According to the divorce‐stress‐adjustment theory (Amato, 2000) and the stress‐buffering model (Wheaton, 1985), personality factors may serve as protective (or risk) factors against the negative effects of divorce on mental health. Indeed, lower neuroticism, indicative of greater emotional stability and likely better coping skills (Wrosch et al., 2003), was associated with lower depression, anxiety, and somatization scores immediately after divorce, supporting the conceptualization of lower neuroticism as a protective factor while positioning higher neuroticism as a risk factor.

However, there is a dearth of research that has examined how personality relates to the longitudinal adjustment to divorce. It is noteworthy that we found that those with higher levels of neuroticism experienced a greater decline in depression, anxiety, and somatization scores, suggesting a more rapid adjustment to the divorce. This finding may seem counter to indications in the literature that those higher in neuroticism may experience more prolonged adjustment issues post‐divorce. However, those higher in neuroticism are assumed to be more emotionally reactive to negative situations (Eysenck & Eysenck, 1975; Lahey, 2009; Mitchell et al., 2021). Thus, it may be that the higher initial scores on depression, anxiety, and somatization reflect the negative initial reaction to a stressful life event at the time of marriage dissolution. In fact, research has consistently suggested that neuroticism is associated with poorer marital satisfaction and higher rates of marital dissolution (Karney & Bradbury, 1995). Moreover, neuroticism has been associated with more negative and maladaptive behaviors during conflict and more hostile attributions for partner's behaviors (Kreuzer & Gollwitzer, 2021). Thus, it may be that those higher in neuroticism react more strongly to marital conflict and the stress of the marital dissolution, resulting in higher initial scores of depression, anxiety, somatization, and stress.

Conversely, the decrease in depression, anxiety, and somatization scores over the 1‐year period after divorce may reflect the removal from a stressful and conflict‐filled situation, and thus, reduced emotional reactivity for those higher in neuroticism. Indeed, increased exposure to conflict may maintain and exacerbate negative emotionality (Joiner & Coyne, 1999), and thus, it may be that for those higher in neuroticism, the reduction in conflict (through reduced contact with the ex‐spouse) may be associated with less negative emotionality and emotional reactivity. Our findings of more elevated levels of psychological distress in terms of symptoms of stress, anxiety, depression, and somatization and a sharper decrease in these symptoms over the first 12 months post‐divorce (i.e., the neuroticism × time interaction), could likely be explained by Lahey's, 2009 hypothesis of emotional reactivity (see Introduction). Further, another study found similar time‐limited moderating effects of neuroticism (Mitchell et al., 2021). However, a longer follow‐up period in our study would be required to confirm the same effects. Alternatively, this stronger decrease in depression, anxiety, and somatization scores may also represent a methodological artifact, in that those with higher initial scores of neuroticism have more “room” to decrease in depression, anxiety, and somatization scores at subsequent time points, relative to those with lower initial scores. This is in line with Kalokerinos et al.’ (2020) conclusions that the relationship between neuroticism and emotional variability is likely a measurement artifact. That is, the measurement and consequent reports of emotional variability are dependent upon the mean, and as people high on neuroticism tend to indicate more severe symptom severity, there is likely a floor‐and ceiling affect.

It is also noteworthy that we did not find consistent support for personality being associated with improved intervention effects. Though some past research suggests that personality, specifically agreeableness, conscientiousness and extraversion (Cerino et al., 2020), and extraversion and openness (Senf & Liau, 2013; Wellenzohn et al., 2018) may moderate intervention effectiveness, the results of our model comparisons echoed those that have found no moderation effect of personality (e.g., Wang et al., 2017). There are several potential explanation for this. For one, these past studies have employed face‐to‐face intervention formats and focused on elderly populations (Cerino et al., 2020), or used positive psychology approaches to the intervention content (Senf & Liau, 2013; Wellenzohn et al., 2018), which may be more effective for certain personality dimensions than others. That is, the relative effectiveness of intervention for certain personality dimensions may be more related to a personality‐intervention fit, as other studies have found that the types of exercises have differential effects depending upon personality (i.e., extraversion) (Schueller, 2012). The CAD divorce intervention took an eclectic approach to intervention content development, relying on several theoretical orientations (Sander et al., in press) as well as more general psychoeducation, which may not target or “favor” any specific personality trait, intervention‐wise.

The current paper represents a first foray into examining the association between personality and longitudinal adjustment to divorce. There are a number of strengths to the current study, including the large sample size and that the sample was obtained close to juridical divorce, enabling us to examine adjustment from the time of the juridical divorce. However, there are also some limitations to consider in interpreting the results. In the current study, we assessed personality 1‐month post‐divorce, and thus, it is unknown whether the divorce and its characteristics may have affected people's responses to the personality questionnaire. Some research suggests that neuroticism is reduced as a function of positive life events (Costa et al., 2000; Mroczek & Spiro, 2003; Neyer & Asendorf, 2001), while neuroticism may increase in response to negative life events, including divorce and the death of a spouse (Koskenvuo et al., 1984; Lockenhoff et al., 2009; Ludtke et al., 2011; Middeldorp et al., 2008; Mroczek & Spiro, 2003). To date, few studies have specifically investigated personality development in relation to divorce, and these studies have typically found few and inconsistent effects of divorce on personality development (Allemand et al., 2015; Asselmann & Specht, 2020; Costa et al., 2000; Roberts & Bogg, 2004; Roberts et al., 2002; Specht et al., 2011), suggesting that any changes may be spurious or idiosyncratic. Further, recent three‐country panel research that followed individuals for up to 6 years concluded that divorce is not a strong nor consistent predictor of changes in personality (Spikic et al., 2021). Thus, we do not have reason to expect that our measurement of personality is biased or inaccurate. It is a limitation, however, that we are unable to control for pre‐divorce—and pre‐intervention—personality. Moreover, the current results should be interpreted in the context of legal divorce; we do not have reliable information regarding whether and for how long participants were no longer residing with their former spouse at the time of the study. Moreover, as participants were recruited at the time of divorce, we were unable to assess and control for pre‐divorce levels of depression, anxiety, somatization, and stress.

A further limitation to note is that the study sample self‐selected into the study. Thus, while the participants were generally representative of the general Danish divorcee population in terms of demographics, it is unknown whether people with higher levels of depression, anxiety, somatization, or stress are overrepresented in the study. Similarly, it is unknown whether people higher or lower on neuroticism self‐selected into the study, or whether participants in the sample scored higher or lower on personality relative to the general Danish population. Future research should seek to examine whether the personality profile of divorcees differs from a general population sample or a married population sample; such information may yield interesting information regarding whether certain personality profiles or facets are associated with divorcing. Moreover, there was a high attrition rate (please also see Hald, Cipric, et al., 2020), and interestingly, attrition analyses suggested that those who dropped out were less extraverted than those who chose to remain in the study. It is unclear why this would be the case. Perhaps divorcees higher in extraversion utilize their social networks more than divorcees lower in extraversion over time, and thus did not need the intervention to the same extent, thus dropping out of the study. In this regard, future research may wish to examine whether and how personality relates to intervention participation.

Finally, it bears mention that neuroticism and anxiety and depression contain many similar facets, suggesting that they may be highly related from a conceptualization standpoint. Indeed, higher neuroticism is marked by a tendency toward more depressed moods and feelings of guilt, envy, anger, and anxiety more frequently and more severely than lower neuroticism (Digman, 1990; Klein et al., 2011). Moreover, people higher in neuroticism tend to be particularly sensitive to situational stress react strongly to conflict and make more hostile attributions for others' behaviors (Fleeson & Gallagher, 2009; Kreuzer & Gollwitzer, 2021; Widiger, 2009). Many of these facets also mark the conceptualizations of depression and anxiety. For instance, individuals who are depressed may also exhibit irritability, mood reactivity, interpersonal sensitivity, and a negative social information processing schema (Fried, 2017). Research on the content overlap between neuroticism and anxiety and depression found that although there was some overlap (e.g., the depressive facet of neuroticism specifically predicted depression symptomology), the relationships between neuroticism and the emotional disorders are not due entirely to content overlap (Uliaszek et al., 2009). In fact, current evidence suggests that depression is linked to the other personality traits as well, such as extraversion/positive emotionality and conscientiousness, therefore suggesting that neuroticism does not entirely explain, but could contribute to the onset and course of depression (Fleeson & Gallagher, 2009; Klein et al., 2011; Markon et al., 2005).

## 5 CONCLUSION

In sum, the current study represents a first attempt to examine whether personality is associated with longitudinal adjustment to divorce, and whether personality moderates the effectiveness of an online divorce intervention. We find that neuroticism is the primary personality factor associated with poor mental health at divorce, but that those higher in neuroticism experience greater symptom improvements as compared with those lower in neuroticism over the 1‐year period post‐divorce. These findings contribute to our understandings of the role of personality, particularly neuroticism, in mental health trajectories after stressful life events, in this case, divorce.

## AUTHOR CONTRIBUTIONS

*Study concept and design*: Gert Martin Hald and Søren Sander. *Analysis and interpretation of data*: Cathrine Lawaetz, Camilla Øverup, Ana Cipric, and Jenna Marie Strizzi. *Drafting of the manuscript*: Cathrine Lawaetz, Camilla Øverup, and Jenna Marie Strizzi. *Critical revision of the manuscript for important intellectual content*: Gert Martin Hald and Ana Cipric. *Statistical analysis*: Camilla Øverup and Jenna Marie Strizzi. *Obtained funding*: Gert Martin Hald and Søren Sander. *Study supervision*: Gert Martin Hald.

## FUNDING INFORMATION

We would like to acknowledge that the Egmont Foundation supported the development of the digital platform “Cooperation After Divorce.” This work was supported by financial support from the Carlsberg Foundation “Distinguished Associate Professor Fellowship” (the last author, prof. Gert Martin Hald) under Grant no. CF16‐0094. Moreover, we thank the Danish State Administration for help during the data collection process

## CONFLICT OF INTEREST

We would like to declare that the University of Copenhagen, Denmark, where the authors work, owns the digital intervention platform “Cooperation after Divorce (CAD),” while two of the co‐authors (Gert Martin Hald and Søren Sander) holds the commercial license and intellectual property rights to the platform through the Company “Cooperation after Divorce” (Samarbejde Efter Skilsmisse, ApS).

## ETHICS APPROVAL

The study followed all ethical and informed consent guidelines throughout the procedure. The study was approved by the Danish Data Protection Agency and the Regional Scientific Ethical Committee of Copenhagen, Denmark.

## PREREGISTRATION

The current study was not pre‐registered prior to data collection or data analysis.

## Supporting information


Supinfo

## Data Availability

The data are not shared within a repository, as consent for such sharing was not obtained from participants at the time of data collection, and the data are of a sensitive nature. To obtain access to the data, please contact the first author via e‐mail to request access.
