# Supplementary material for: Mental health trajectories after juridical divorce: Does personality matter?
Source: J Pers. 2022 Jun 22;91(2):426–40. doi: 10.1111/jopy.12737 (PMC10083938; doi:10.1111/jopy.12737)
Supplement: Supplementary file 1 — Supinfo [file JOPY-91-426-s001.docx]

Table 2

*Correlations between Personality Dimensions, Depression, Anxiety, Somatization, and Stress (Part 1)*

|  |  |  |  |  | 5. | 6. | 7. | 8. | 9. | 10. | 11. | 12. |
| --- | --- | --- | --- | --- | --- | --- | --- | --- | --- | --- | --- | --- |
| 1. Neuroticism | - |  |  |  |  |  |  |  |  |  |  |  |
| 2. Extraversion | -.519** | - |  |  |  |  |  |  |  |  |  |  |
| 3. Openness | -0.070 | .366** | - |  |  |  |  |  |  |  |  |  |
| 4. Agreeableness | -0.021 | -0.010 | .144** | - |  |  |  |  |  |  |  |  |
| 5. Conscientiousness | -.524** | .312** | 0.071 | .186** | - |  |  |  |  |  |  |  |
| 6. Anxiety (BL) | .508** | -.217** | 0.035 | 0.047 | -.173** | - |  |  |  |  |  |  |
| 7. Depression (BL) | .558** | -.291** | -.084* | 0.064 | -.239** | .810** | - |  |  |  |  |  |
| 8. Somatization (BL) | .445** | -.206** | -0.005 | 0.061 | -.177** | .777** | .678** | - |  |  |  |  |
| 9. Stress (BL) | .564** | -.246** | -.030 | .016 | -.276** | .694** | .775** | .569** | - |  |  |  |
| 10. Anxiety (3) | .548** | -.299** | 0.091 | .116* | -.211** | .706** | .609** | .595** | .528** | - |  |  |
| 11. Depression (3) | .546** | -.373** | -0.031 | .161** | -.234** | .613** | .702** | .541** | .550** | .832** | - |  |
| 12. Somatization (3) | .436** | -.258** | 0.009 | .116* | -.184** | .560** | .528** | .711** | .475** | .756** | .702** | - |
| 13. Stress (3) | .607** | -.358** | -.026 | .128* | -.277** | .532** | .600** | .459** | .616** | .745** | .831** | .592** |
| 14. Anxiety (6) | .428** | -.168** | .158** | 0.038 | -.140* | .569** | .447** | .429** | .466** | .690** | .551** | .568** |
| 15. Depression (6) | .502** | -.255** | 0.039 | 0.053 | -.234** | .497** | .554** | .399** | .531** | .602** | .705** | .506** |
| 16. Somatization (6) | .357** | -.154** | 0.035 | 0.098 | -.165** | .470** | .404** | .607** | .431** | .579** | .531** | .748** |
| 17. Stress (6) | .543** | -.222** | .007 | .037 | -.263** | .394** | .434** | .358** | .546** | .628** | .659** | .545** |
| 18. Anxiety (12) | .417** | -.244** | 0.037 | 0.016 | -.213** | .581** | .446** | .494** | .443** | .605** | .535** | .585** |
| 19. Depression (12) | .491** | -.303** | -0.091 | 0.035 | -.247** | .500** | .562** | .431** | .524** | .562** | .697** | .571** |
| 20. Somatization (12) | .308** | -.177** | -0.038 | 0.043 | -.150* | .450** | .383** | .597** | .385** | .422** | .418** | .711** |
| 21. Stress (12) | .500** | -.245** | -.090 | -.040 | -.307** | .437** | .476** | .438** | .536** | .557** | .579** | .558** |
| 22. Gender | .159** | -0.019 | .084* | .245** | .087* | .071** | .064** | .172** | .100** | .111* | .118** | .194** |
| 23. Educational level | -.145** | .140** | .231** | -0.027 | 0.056 | -.159** | -.182** | -.164** | -.093* | -.135** | -.138** | -.206** |
| 24. Income | -.284** | .205** | 0.045 | -.122** | .160** | -.167** | -.185** | -.202** | -.162** | -.212** | -.229** | -.232** |

*Note*. * *p* < .05, ** *p* < .001. (BL) = at baseline, (3) = at 3 months, (6) = at 6 months, (12) = at 12 months

Table 2

*Correlations between Personality Dimensions, Depression, Anxiety, Somatization, and Stress (Part 2)*

|  | 13 | 14 | 15 | 16 | 17 | 18 | 19 | 20 | 21 | 22 | 23 |
| --- | --- | --- | --- | --- | --- | --- | --- | --- | --- | --- | --- |
| 1. Neuroticism |  |  |  |  |  |  |  |  |  |  |  |
| 2. Extraversion |  |  |  |  |  |  |  |  |  |  |  |
| 3. Openness |  |  |  |  |  |  |  |  |  |  |  |
| 4. Agreeableness |  |  |  |  |  |  |  |  |  |  |  |
| 5. Conscientiousness |  |  |  |  |  |  |  |  |  |  |  |
| 6. Anxiety (BL) |  |  |  |  |  |  |  |  |  |  |  |
| 7. Depression (BL) |  |  |  |  |  |  |  |  |  |  |  |
| 8. Somatization (BL) |  |  |  |  |  |  |  |  |  |  |  |
| 9. Stress (BL) |  |  |  |  |  |  |  |  |  |  |  |
| 10. Anxiety (3) |  |  |  |  |  |  |  |  |  |  |  |
| 11. Depression (3) |  |  |  |  |  |  |  |  |  |  |  |
| 12. Somatization (3) |  |  |  |  |  |  |  |  |  |  |  |
| 13. Stress (3) | - |  |  |  |  |  |  |  |  |  |  |
| 14. Anxiety (6) | .555** | - |  |  |  |  |  |  |  |  |  |
| 15. Depression (6) | .636** | .796** | - |  |  |  |  |  |  |  |  |
| 16. Somatization (6) | .524** | .743** | .681** | - |  |  |  |  |  |  |  |
| 17. Stress (6) | .736** | .706** | .792** | .623** | - |  |  |  |  |  |  |
| 18. Anxiety (12) | .443** | .653** | .572** | .638** | .570** | - |  |  |  |  |  |
| 19. Depression (12) | .557** | .556** | .700** | .540** | .644** | .804** | - |  |  |  |  |
| 20. Somatization (12) | .320** | .499** | .447** | .714** | .426** | .751** | .662** | - |  |  |  |
| 21. Stress (12) | .637** | .512** | .614** | .505** | .701** | .717** | .812** | .579** | - |  |  |
| 22. Gender | .189** | .128** | 0.075 | .191** | .141* | .160** | .134** | .164** | .151* | - |  |
| 23. Educational level | -.057 | -0.080 | -.121* | -.108* | -.053 | -.119* | -.137** | -.157** | -.109 | .067** | - |
| 24. Income | -.220** | -.136** | -.198** | -.179** | -.147** | -.173** | -.173** | -.215** | -.123* | -.243** | .316** |

*Note*. * *p* < .05, ** *p* < .001. (BL) = at baseline, (3) = at 3 months, (6) = at 6 months, (12) = at 12 months

Table 4s

*Results of Linear Mixed Effect Modeling to assess the associations between all five personality dimensions and anxiety, depression, somatization symptoms and stress scores adjusted for gender, educational level, and income*

|  | Model 4^a^ | | | | Model 3^b^ | | | | Model 2^c^ | | | | Model 1^d^ | | | |
| --- | --- | --- | --- | --- | --- | --- | --- | --- | --- | --- | --- | --- | --- | --- | --- | --- |
| Variable | Estimate | SE | Cohen’s d | *p* | Estimate | SE | Cohen’s d | *p* | Estimate | SE | Cohen’s d | *p* | Estimate | SE | Cohen’s d | *p* |
| **Anxiety** | | | | | | | | | | | | | | | | |
| Intercept | **0.8991** | **0.125** | **2.054** | **<.001** | **0.901** | **0.126** | **2.055** | **<.001** | **0.890** | **0.125** | **2.047** | **<.001** | **0.926** | **0.140** | **1.687** | **<.001** |
| Time | **-0.041** | **0.009** | **-0.093** | **<.001** | **-0.041** | **0.009** | **-0.092** | **<.001** | **-0.041** | **0.008** | **-0.094** | **<.001** | **-0.040** | **0.008** | **-0.073** | **<.001** |
| Group | -0.004 | 0.044 | -0.009 | 0.926 | -0.006 | 0.044 | -0.014 | 0.894 | 0.001 | 0.045 | 0.003 | 0.980 | 0.042 | 0.051 | 0.076 | 0.417 |
| Neuroticism | **0.055** | **0.005** | **0.125** | **<.001** | **0.052** | **0.005** | **0.119** | **<.001** | **0.045** | **0.004** | **0.103** | **<.001** |  |  |  |  |
| Extraversion | 0.008 | 0.005 | 0.019 | 0.111 | 0.007 | 0.005 | 0.017 | 0.146 | 0.005 | 0.005 | 0.011 | 0.329 |  |  |  |  |
| Openness | 0.003 | 0.005 | 0.007 | 0.569 | 0.003 | 0.005 | 0.006 | 0.598 | 0.003 | 0.005 | 0.008 | 0.481 |  |  |  |  |
| Agreeableness | -0.001 | 0.005 | -0.002 | 0.847 | -0.000 | 0.005 | -0.000 | 0.950 | -0.002 | 0.005 | -0.005 | 0.663 |  |  |  |  |
| Conscientiousness | **0.016** | **0.006** | **0.038** | **0.005** | **0.016** | **0.006** | **0.036** | **0.006** | **0.012** | **0.005** | **0.029** | **0.022** |  |  |  |  |
| Group*Neuroticism | -0.010 | 0.007 | -0.022 | 0.148 | -0.004 | 0.006 | -0.010 | 0.480 | -0.005 | 0.006 | -0.011 | 0.425 |  |  |  |  |
| Group*Extraversion | -0.015 | 0.008 | -0.034 | 0.056 | -0.013 | 0.007 | -0.029 | 0.074 | -0.013 | 0.007 | -0.031 | 0.061 |  |  |  |  |
| Group*Openness | 0.014 | 0.008 | 0.032 | 0.074 | **0.015** | **0.007** | **0.033** | **0.041** | **0.015** | **0.007** | **0.034** | **0.041** |  |  |  |  |
| Group*Agreeableness | 0.012 | 0.008 | 0.027 | 0.160 | 0.010 | 0.008 | 0.022 | 0.206 | 0.009 | 0.008 | 0.021 | 0.239 |  |  |  |  |
| Group*Conscientiousness | -0.005 | 0.009 | -0.012 | 0.555 | -0.003 | 0.008 | -0.008 | 0.679 | -0.003 | 0.008 | -0.006 | 0.761 |  |  |  |  |
| Time*Neuroticism | **-0.003** | **0.001** | **-0.007** | **<.001** | **-0.002** | **0.000** | **-0.006** | **<.001** |  |  |  |  |  |  |  |  |
| Time*Extraversion | -0.001 | 0.001 | -0.002 | 0.076 | -0.001 | 0.000 | -0.002 | 0.075 |  |  |  |  |  |  |  |  |
| Time*Openness | 0.000 | 0.001 | 0.000 | 0.928 | 0.000 | 0.000 | 0.000 | 0.712 |  |  |  |  |  |  |  |  |
| Time*Agreeableness | -0.000 | 0.001 | -0.001 | 0.554 | -0.001 | 0.000 | -0.001 | 0.211 |  |  |  |  |  |  |  |  |
| Time*Conscientiousness | -0.001 | 0.001 | -0.003 | 0.105 | -0.001 | 0.001 | -0.002 | 0.094 |  |  |  |  |  |  |  |  |
| Time*Group*Neuroticism | **0.002** | **0.001** | **0.004** | **0.038** |  |  |  |  |  |  |  |  |  |  |  |  |
| Time*Group*Extraversion | 0.001 | 0.001 | 0.001 | 0.519 |  |  |  |  |  |  |  |  |  |  |  |  |
| Time*Group*Openness | 0.000 | 0.001 | 0.001 | 0.765 |  |  |  |  |  |  |  |  |  |  |  |  |
| Time*Group*Agreeableness | -0.001 | 0.001 | -0.001 | 0.526 |  |  |  |  |  |  |  |  |  |  |  |  |
| Time*Group*Conscientiousness | 0.001 | 0.001 | 0.001 | 0.572 |  |  |  |  |  |  |  |  |  |  |  |  |
| Time*Group | **0.031** | **0.005** | **0.070** | **<.001** | **0.031** | **0.005** | **0.071** | **<.001** | **0.029** | **0.005** | **0.067** | **<.001** | **0.029** | **0.005** | **0.053** | **<.001** |
| Gender | -0.010 | 0.047 | -0.024 | 0.827 | -0.012 | 0.047 | -0.027 | 0.803 | -0.005 | 0.047 | -0.011 | 0.922 | 0.102 | 0.053 | 0.186 | 0.054 |
| Education | -0.006 | 0.012 | -0.014 | 0.608 | -0.006 | 0.012 | -0.014 | 0.622 | -0.007 | 0.012 | -0.016 | 0.573 | -0.013 | 0.014 | -0.024 | 0.341 |
| Income | -0.009 | 0.013 | -0.021 | 0.498 | -0.010 | 0.013 | -0.022 | 0.475 | -0.008 | 0.013 | -0.020 | 0.524 | **-0.053** | **0.015** | **-0.096** | **0.001** |
| **Depression** | | | | | | | | | | | | | | | | |
|  | Model 4^a^ | | | | Model 3^b^ | | | | Model 2^c^ | | | | Model 1^d^ | | | |
|  | Estimate | SE | Cohen’s d | *p* | Estimate | SE | Cohen’s d | *p* | Estimate | SE | Cohen’s d | *p* | Estimate | SE | Cohen’s d | *p* |
| Intercept | **1.446** | **0.159** | **2.726** | **<.001** | **1.447** | **0.160** | **2.726** | **<.001** | **1.438** | **0.159** | **2.731** | **<.001** | **1.608** | **0.182** | **2.304** | **<.001** |
| Time | **-0.063** | **0.012** | **-0.118** | **<.001** | **-0.062** | **0.012** | **-0.117** | **<.001** | **-0.063** | **0.012** | **-0.119** | **<.001** | **-0.061** | **0.011** | **-0.088** | **<.001** |
| Group | -0.001 | 0.056 | -0.001 | 0.989 | -0.003 | 0.056 | -0.006 | 0.954 | 0.001 | 0.056 | 0.002 | 0.986 | 0.052 | 0.066 | 0.075 | 0.429 |
| Neuroticism | **0.066** | **0.006** | **0.125** | **<.001** | **0.061** | **0.006** | **0.115** | **<.001** | **0.055** | **0.006** | **0.104** | **<.001** |  |  |  |  |
| Extraversion | 0.009 | 0.007 | 0.017 | 0.171 | 0.005 | 0.006 | 0.009 | 0.426 | 0.002 | 0.006 | 0.004 | 0.709 |  |  |  |  |
| Openness | -0.010 | 0.007 | -0.018 | 0.143 | -0.009 | 0.006 | -0.017 | 0.168 | -0.006 | 0.006 | -0.012 | 0.304 |  |  |  |  |
| Agreeableness | 0.003 | 0.007 | 0.006 | 0.621 | 0.005 | 0.007 | 0.010 | 0.444 | 0.001 | 0.006 | 0.001 | 0.903 |  |  |  |  |
| Conscientiousness | 0.010 | 0.007 | 0.018 | 0.192 | 0.009 | 0.007 | 0.017 | 0.216 | 0.007 | 0.007 | 0.014 | 0.289 |  |  |  |  |
| Group*Neuroticism | -0.009 | 0.008 | -0.017 | 0.288 | 0.001 | 0.008 | 0.003 | 0.855 | 0.001 | 0.008 | 0.002 | 0.912 |  |  |  |  |
| Group*Extraversion | **-0.022** | **0.010** | **-0.042** | **0.022** | -0.014 | 0.009 | -0.026 | 0.125 | -0.014 | 0.009 | -0.027 | 0.110 |  |  |  |  |
| Group*Openness | 0.012 | 0.010 | 0.024 | 0.203 | 0.011 | 0.009 | 0.020 | 0.230 | 0.011 | 0.009 | 0.020 | 0.233 |  |  |  |  |
| Group*Agreeableness | **0.024** | **0.011** | **0.045** | **0.024** | **0.019** | **0.010** | **0.037** | **0.044** | 0.019 | 0.010 | 0.035 | 0.054 |  |  |  |  |
| Group*Conscientiousness | -0.005 | 0.011 | -0.009 | 0.662 | -0.004 | 0.010 | -0.007 | 0.733 | -0.003 | 0.010 | -0.005 | 0.781 |  |  |  |  |
| Time*Neuroticism | **-0.004** | **0.001** | **-0.007** | **<.001** | **-0.002** | **0.001** | **-0.004** | **<.001** |  |  |  |  |  |  |  |  |
| Time*Extraversion | **-0.002** | **0.001** | **-0.004** | **0.012** | -0.001 | 0.001 | -0.002 | 0.162 |  |  |  |  |  |  |  |  |
| Time*Openness | 0.001 | 0.001 | 0.002 | 0.246 | 0.001 | 0.001 | 0.001 | 0.236 |  |  |  |  |  |  |  |  |
| Time*Agreeableness | -0.001 | 0.001 | -0.002 | 0.315 | **-0.001** | **0.001** | **-0.003** | **0.033** |  |  |  |  |  |  |  |  |
| Time*Conscientiousness | -0.001 | 0.001 | -0.001 | 0.442 | -0.000 | 0.001 | -0.001 | 0.538 |  |  |  |  |  |  |  |  |
| Time*Group*Neuroticism | **0.003** | **0.001** | **0.006** | **0.003** |  |  |  |  |  |  |  |  |  |  |  |  |
| Time*Group*Extraversion | **0.003** | **0.001** | **0.005** | **0.033** |  |  |  |  |  |  |  |  |  |  |  |  |
| Time*Group*Openness | -0.000 | 0.001 | -0.001 | 0.719 |  |  |  |  |  |  |  |  |  |  |  |  |
| Time*Group*Agreeableness | -0.001 | 0.001 | -0.003 | 0.300 |  |  |  |  |  |  |  |  |  |  |  |  |
| Time*Group*Conscientiousness | 0.001 | 0.001 | 0.001 | 0.729 |  |  |  |  |  |  |  |  |  |  |  |  |
| Time*Group | **0.049** | **0.007** | **0.093** | **<.001** | **0.050** | **0.007** | **0.093** | **<.001** | **0.048** | **0.007** | **0.092** | **<.001** | **0.048** | **0.007** | **0.069** | **<.001** |
| Gender | 0.013 | 0.059 | 0.024 | 0.829 | 0.011 | 0.059 | 0.020 | 0.855 | 0.017 | 0.059 | 0.032 | 0.777 | **0.145** | **0.068** | **0.208** | **0.033** |
| Education | -0.014 | 0.015 | -0.027 | 0.346 | -0.014 | 0.015 | -0.026 | 0.372 | -0.015 | 0.015 | -0.028 | 0.336 | -0.035 | 0.018 | -0.050 | 0.053 |
| Income | -0.000 | 0.017 | -0.001 | 0.986 | -0.001 | 0.017 | -0.002 | 0.947 | 0.000 | 0.017 | 0.001 | 0.985 | **-0.066** | **0.020** | **-0.095** | **<.001** |
| **Somatization** | | | | | | | | | | | | | | | | |
|  | Model 4^a^ | | | | Model 3^b^ | | | | Model 2^c^ | | | | Model 1^d^ | | | |
|  | Estimate | SE | Cohen’s d | *p* | Estimate | SE | Cohen’s d | *p* | Estimate | SE | Cohen’s d | *p* | Estimate | SE | Cohen’s d | *p* |
| Intercept | **0.654** | **0.118** | **1.38** | **<.001** | **0.654** | **0.118** | **1.378** | **<.001** | **0.647** | **0.118** | **1.361** | **<.001** | **0.712** | **0.125** | **1.341** | **<.001** |
| Time | **-0.027** | **0.005** | **-0.056** | **<.001** | **-0.026** | **0.005** | **-0.055** | **<.001** | **-0.026** | **0.004** | **-0.055** | **<.001** | **-0.026** | **0.004** | **-0.048** | **<.001** |
| Group | 0.029 | 0.044 | 0.062 | 0.510 | 0.030 | 0.044 | 0.063 | 0.504 | 0.035 | 0.045 | 0.074 | 0.432 | 0.063 | 0.048 | 0.119 | 0.188 |
| Neuroticism | **0.036** | **0.005** | **0.077** | **<.001** | **0.036** | **0.005** | **0.076** | **<.001** | **0.031** | **0.005** | **0.066** | **<.001** |  |  |  |  |
| Extraversion | 0.005 | 0.005 | 0.010 | 0.356 | 0.004 | 0.005 | 0.009 | 0.381 | 0.003 | 0.005 | 0.006 | 0.529 |  |  |  |  |
| Openness | 0.000 | 0.005 | 0.000 | 0.990 | 0.000 | 0.005 | 0.000 | 0.972 | 0.000 | 0.005 | 0.001 | 0.954 |  |  |  |  |
| Agreeableness | 0.000 | 0.005 | 0.001 | 0.937 | 0.001 | 0.005 | 0.002 | 0.858 | 0.000 | 0.005 | 0.000 | 0.968 |  |  |  |  |
| Conscientiousness | 0.007 | 0.006 | 0.015 | 0.216 | 0.008 | 0.006 | 0.018 | 0.146 | 0.007 | 0.006 | 0.015 | 0.202 |  |  |  |  |
| Group*Neuroticism | -0.002 | 0.007 | -0.005 | 0.714 | -0.002 | 0.006 | -0.004 | 0.783 | -0.002 | 0.006 | -0.005 | 0.732 |  |  |  |  |
| Group*Extraversion | -0.007 | 0.008 | -0.015 | 0.342 | -0.006 | 0.007 | -0.013 | 0.389 | -0.007 | 0.007 | -0.014 | 0.369 |  |  |  |  |
| Group*Openness | 0.004 | 0.008 | 0.008 | 0.636 | 0.003 | 0.007 | 0.007 | 0.649 | 0.003 | 0.007 | 0.007 | 0.665 |  |  |  |  |
| Group*Agreeableness | 0.010 | 0.008 | 0.022 | 0.211 | 0.009 | 0.008 | 0.020 | 0.232 | 0.009 | 0.008 | 0.019 | 0.254 |  |  |  |  |
| Group*Conscientiousness | -0.006 | 0.009 | -0.014 | 0.467 | -0.009 | 0.008 | -0.019 | 0.289 | -0.008 | 0.008 | -0.018 | 0.325 |  |  |  |  |
| Time*Neuroticism | **-0.002** | **0.000** | **-0.003** | **<.001** | **-0.001** | **0.000** | **-0.003** | **<.001** |  |  |  |  |  |  |  |  |
| Time*Extraversion | -0.000 | 0.001 | -0.001 | 0.349 | -0.000 | 0.000 | -0.001 | 0.299 |  |  |  |  |  |  |  |  |
| Time*Openness | -0.000 | 0.001 | -0.000 | 0.979 | -0.000 | 0.000 | -0.000 | 0.955 |  |  |  |  |  |  |  |  |
| Time*Agreeableness | -0.000 | 0.001 | -0.000 | 0.873 | -0.000 | 0.000 | -0.001 | 0.558 |  |  |  |  |  |  |  |  |
| Time*Conscientiousness | 0.000 | 0.001 | 0.000 | 0.931 | -0.000 | 0.000 | -0.001 | 0.524 |  |  |  |  |  |  |  |  |
| Time*Group*Neuroticism | 0.000 | 0.001 | 0.000 | 0.772 |  |  |  |  |  |  |  |  |  |  |  |  |
| Time*Group*Extraversion | 0.000 | 0.001 | 0.001 | 0.679 |  |  |  |  |  |  |  |  |  |  |  |  |
| Time*Group*Openness | -0.000 | 0.001 | -0.000 | 0.921 |  |  |  |  |  |  |  |  |  |  |  |  |
| Time*Group*Agreeableness | -0.000 | 0.001 | -0.001 | 0.713 |  |  |  |  |  |  |  |  |  |  |  |  |
| Time*Group*Conscientiousness | -0.001 | 0.001 | -0.002 | 0.374 |  |  |  |  |  |  |  |  |  |  |  |  |
| Time*Group | **0.0237** | **0.005** | **0.050** | **<.001** | **0.023** | **0.005** | **0.049** | **<.001** | **0.022** | **0.005** | **0.046** | **<.001** | **0.022** | **0.005** | **0.041** | **<.001** |
| Gender | **0.142** | **0.048** | **0.299** | **0.003** | **0.140** | **0.048** | **0.296** | **0.004** | **0.145** | **0.048** | **0.305** | **0.003** | **0.221** | **0.050** | **0.416** | **<.001** |
| Education | -0.016 | 0.013 | -0.033 | 0.213 | -0.015 | 0.013 | -0.032 | 0.226 | -0.016 | 0.013 | -0.033 | 0.209 | -0.025 | 0.013 | -0.048 | 0.057 |
| Income | -0.019 | 0.014 | -0.039 | 0.171 | -0.019 | 0.014 | -0.039 | 0.170 | -0.018 | 0.014 | -0.038 | 0.186 | **-0.052** | **0.014** | **-0.098** | **<.001** |
| **Stress** | | | | | | | | | | | | | | | | |
|  | Model 4^a^ | | | | Model 3^b^ | | | | Model 2^c^ | | | | Model 1^d^ | | | |
|  | Estimate | SE | Cohen’s d | *p* | Estimate | SE | Cohen’s d | *p* | Estimate | SE | Cohen’s d | *p* | Estimate | SE | Cohen’s d | *p* |
| Intercept | **17.78** | **1.401** | **4.588** | **<.001** | **17.79** | **1.405** | **4.578** | **<.001** | **17.72** | **1.392** | **4.583** | **<.001** | **19.2** | **1.56** | **3.595** | **<.001** |
| Time | **-0.573** | **0.138** | **-0.148** | **<.001** | **-0.569** | **0.139** | **-0.147** | **<.001** | **-0.573** | **0.136** | **-0.148** | **<.001** | **-0.562** | **0.133** | **-0.105** | **<.001** |
| Group | -0.063 | 0.426 | -0.016 | 0.883 | -0.077 | 0.427 | -0.020 | 0.858 | -0.035 | 0.426 | -0.009 | 0.934 | 0.423 | 0.512 | 0.079 | 0.408 |
| Neuroticism | **0.537** | **0.046** | **0.138** | **<.001** | **0.501** | **0.044** | **0.129** | **<.001** | **0.463** | **0.042** | **0.120** | **<.001** |  |  |  |  |
| Extraversion | **0.131** | **0.049** | **0.034** | **0.008** | 0.088 | 0.047 | 0.023 | 0.063 | 0.085 | 0.045 | 0.022 | 0.057 |  |  |  |  |
| Openness | -0.074 | 0.051 | -0.019 | 0.146 | -0.062 | 0.049 | -0.016 | 0.204 | -0.083 | 0.046 | -0.022 | 0.069 |  |  |  |  |
| Agreeableness | 0.034 | 0.052 | 0.009 | 0.506 | 0.031 | 0.05 | 0.008 | 0.539 | 0.014 | 0.047 | 0.004 | 0.767 |  |  |  |  |
| Conscientiousness | 0.051 | 0.056 | 0.013 | 0.363 | 0.045 | 0.054 | 0.012 | 0.403 | 0.016 | 0.051 | 0.004 | 0.752 |  |  |  |  |
| Group*Neuroticism | -0.085 | 0.064 | -0.022 | 0.185 | -0.013 | 0.058 | -0.003 | 0.828 | -0.014 | 0.058 | -0.004 | 0.806 |  |  |  |  |
| Group*Extraversion | -0.187 | 0.074 | -0.048 | 0.011 | -0.092 | 0.066 | -0.024 | 0.168 | -0.094 | 0.066 | -0.024 | 0.156 |  |  |  |  |
| Group*Openness | **0.152** | **0.074** | **0.039** | **0.042** | 0.130 | 0.067 | 0.033 | 0.053 | 0.132 | 0.067 | 0.034 | 0.050 |  |  |  |  |
| Group*Agreeableness | 0.076 | 0.080 | 0.020 | 0.339 | 0.083 | 0.072 | 0.021 | 0.249 | 0.078 | 0.072 | 0.020 | 0.280 |  |  |  |  |
| Group*Conscientiousness | -0.068 | 0.086 | -0.018 | 0.428 | -0.064 | 0.078 | -0.017 | 0.407 | -0.056 | 0.078 | -0.015 | 0.467 |  |  |  |  |
| Time*Neuroticism | **-0.023** | **0.006** | **-0.006** | **0.000** | **-0.012** | **0.004** | **-0.003** | **0.005** |  |  |  |  |  |  |  |  |
| Time*Extraversion | **-0.014** | **0.006** | **-0.004** | **0.030** | -0.001 | 0.005 | -0.000 | 0.850 |  |  |  |  |  |  |  |  |
| Time*Openness | -0.003 | 0.007 | -0.001 | 0.659 | -0.006 | 0.005 | -0.002 | 0.191 |  |  |  |  |  |  |  |  |
| Time*Agreeableness | -0.007 | 0.007 | -0.002 | 0.311 | -0.005 | 0.005 | -0.001 | 0.284 |  |  |  |  |  |  |  |  |
| Time*Conscientiousness | -0.010 | 0.007 | -0.002 | 0.182 | -0.008 | 0.006 | -0.002 | 0.166 |  |  |  |  |  |  |  |  |
| Neuroticism*Time*Group | **0.021** | **0.008** | **0.005** | **0.012** |  |  |  |  |  |  |  |  |  |  |  |  |
| Time*Group*Extraversion | **0.027** | **0.009** | **0.007** | **0.003** |  |  |  |  |  |  |  |  |  |  |  |  |
| Time*Group*Openness | -0.006 | 0.010 | -0.002 | 0.556 |  |  |  |  |  |  |  |  |  |  |  |  |
| Time*Group*Agreeableness | 0.002 | 0.010 | 0.001 | 0.849 |  |  |  |  |  |  |  |  |  |  |  |  |
| Time*Group*Conscientiousness | 0.001 | 0.011 | 0.000 | 0.917 |  |  |  |  |  |  |  |  |  |  |  |  |
| Time*Group | **0.454** | **0.055** | **0.117** | **<.001** | **0.454** | **0.054** | **0.117** | **<.001** | **0.440** | **0.054** | **0.114** | **<.001** | **0.437** | **0.055** | **0.082** | **<.001** |
| Gender | 0.298 | 0.440 | 0.077 | 0.499 | 0.287 | 0.441 | 0.074 | 0.515 | 0.316 | 0.440 | 0.082 | 0.473 | **1.188** | **0.524** | **0.222** | **0.023** |
| Education | 0.025 | 0.115 | 0.006 | 0.827 | 0.028 | 0.115 | 0.007 | 0.808 | 0.029 | 0.115 | 0.007 | 0.803 | -0.120 | 0.140 | -0.023 | 0.389 |
| Income | 0.01797 | 0.125 | 0.005 | 0.885 | 0.015 | 0.125 | 0.004 | 0.901 | 0.021 | 0.125 | 0.005 | 0.866 | **-0.498** | **0.151** | **-0.093** | **<.001** |

*Note.*

^a^ Model 4 assessed for a group by personality by time interaction

^b^ Model 3 assessed for a personality by time interaction

^c^ Model 2 assessed for the main effect of personality

^d^ Model 1 assessed assumes no effect of personality

Table 4

*Results of Linear Mixed Effect Modeling to assess the associations between the neuroticism personality dimensions and anxiety, depression, somatization symptoms and stress scores adjusted for gender, educational level, and income*

|  | Model 4^a^ | | | | Model 3^b^ | | | | Model 2^c^ | | | | Model 1^d^ | | | |
| --- | --- | --- | --- | --- | --- | --- | --- | --- | --- | --- | --- | --- | --- | --- | --- | --- |
| Variable | Estimate | SE | Cohen’s d | *p* | Estimate | SE | Cohen’s d | *p* | Estimate | SE | Cohen’s d | *p* | Estimate | SE | Cohen’s d | *p* |
| Anxiety | | | | | | | | | | | | | | | | |
| (Intercept) | **0.796** | **0.124** | **1.783** | **< .001** | **0.798** | **0.124** | **1.784** | **< .001** | **0.788** | **0.1231** | **1.775** | **< .001** | **0.9255** | **0.140** | **1.687** | **< .001** |
| Time | **-0.041** | **0.008** | **-0.092** | **< .001** | **-0.041** | **0.008** | **-0.092** | **< .001** | **-0.041** | **0.008** | **-0.092** | **< .001** | **-0.040** | **0.008** | **-0.073** | **< .001** |
| Group | -0.009 | 0.044 | -0.020 | 0.840 | -0.010 | 0.045 | -0.023 | 0.828 | -0.001 | 0.045 | -0.003 | 0.974 | 0.042 | 0.051 | 0.076 | 0.417 |
| Neuroticism | **0.045** | **0.004** | **0.101** | **< .001** | **0.043** | **0.004** | **0.097** | **< .001** | **0.038** | **0.003** | **0.085** | **< .001** |  |  |  |  |
| Group*Neuroticism | -0.002 | 0.005 | -0.005 | 0.663 | 0.001 | 0.005 | 0.003 | 0.758 | 0.001 | 0.005 | 0.002 | 0.869 |  |  |  |  |
| Time*Neuroticism | **0.031** | **0.000** | **-0.005** | **< .001** | **-0.002** | **0.000** | **-0.004** | **< .001** |  |  |  |  |  |  |  |  |
| Time*Group*Neuroticism | 0.035 | 0.001 | 0.003 | 0.056 |  |  |  |  |  |  |  |  |  |  |  |  |
| Time*Group | **0.001** | **0.005** | **0.070** | **< .001** | **0.0317** | **0.005** | **0.071** | **< .001** | **0.030** | **0.005** | **0.067** | **< .001** | **0.029** | **0.005** | **0.053** | **< .001** |
| Gender | -0.002 | 0.045 | 0.0790 | 0.438 | 0.034 | 0.045 | 0.075 | 0.459 | 0.039 | 0.045 | 0.088 | 0.389 | 0.102 | 0.053 | 0.186 | 0.054 |
| Education | -0.008 | 0.012 | -0.003 | 0.897 | -0.001 | 0.012 | -0.003 | 0.914 | -0.002 | 0.012 | -0.005 | 0.845 | -0.013 | 0.014 | -0.024 | 0.341 |
| Income | -0.008 | 0.013 | -0.018 | 0.546 | -0.008 | 0.013 | -0.019 | 0.534 | -0.007 | 0.013 | -0.016 | 0.590 | **-0.053** | **0.015** | **-0.096** | **< .001** |
| Depression |  |  |  |  |  |  |  |  |  |  |  |  |  |  |  |  |
| (Intercept) | **1.423** | **0.156** | **2.672** | **< .001** | **1.425** | **0.156** | **2.677** | **< .001** | **1.417** | **0.155** | **2.674** | **< .001** | **1.608** | **0.182** | **2.304** | **< .001** |
| Time | **-0.063** | **0.012** | **-0.118** | **< .001** | **-0.063** | **0.012** | **-0.118** | **< .001** | **-0.063** | **0.011** | **-0.118** | **< .001** | **-0.061** | **0.011** | **-0.088** | **< .001** |
| Group | -0.017 | 0.056 | -0.032 | 0.761 | -0.018 | 0.056 | -0.034 | 0.745 | -0.011 | 0.056 | -0.021 | 0.846 | 0.052 | 0.066 | 0.075 | 0.429 |
| Neuroticism | **0.059** | **0.005** | **0.110** | **< .001** | **0.056** | **0.004** | **0.105** | **< .001** | **0.051** | **0.004** | **0.096** | **< .001** |  |  |  |  |
| Group*Neuroticism | 0.002 | 0.006 | 0.004 | 0.722 | 0.008 | 0.006 | 0.015 | 0.152 | 0.008 | 0.006 | 0.015 | 0.184 |  |  |  |  |
| Time*Neuroticism | **-0.003** | **0.001** | **-0.005** | **< .001** | **-0.002** | **0.000** | **-0.003** | **< .001** |  |  |  |  |  |  |  |  |
| Time*Group*Neuroticism | **0.002** | **0.001** | **0.004** | **0.019** |  |  |  |  |  |  |  |  |  |  |  |  |
| Time*Group | **0.050** | **0.007** | **0.094** | **< .001** | **0.051** | **0.007** | **0.095** | **< .001** | **0.049** | **0.007** | **0.092** | **< .001** | **0.048** | **0.007** | **0.069** | **< .001** |
| Gender | 0.051 | 0.056 | 0.097 | 0.359 | 0.049 | 0.056 | 0.092 | 0.383 | 0.053 | 0.056 | 0.101 | 0.341 | **0.145** | **0.068** | **0.208** | **0.033** |
| Education | -0.019 | 0.015 | -0.035 | 0.208 | -0.018 | 0.015 | -0.035 | 0.217 | -0.019 | 0.015 | -0.036 | 0.195 | -0.035 | 0.018 | -0.050 | 0.053 |
| Income | -0.002 | 0.016 | -0.004 | 0.896 | -0.003 | 0.016 | -0.005 | 0.877 | -0.002 | 0.016 | -0.003 | 0.919 | **-0.066** | **0.020** | **-0.095** | **< .001** |
| Somatization |  |  |  |  |  |  |  |  |  |  |  |  |  |  |  |  |
| (Intercept) | **0.619** | **0.115** | **1.308** | **< .001** | **0.619** | **0.115** | **1.309** | **< .001** | **0.614** | **0.115** | **1.295** | **< .001** | **0.712** | **0.125** | **1.341** | **< .001** |
| Time | **-0.026** | **0.005** | **-0.056** | **< .001** | **-0.026** | **0.005** | **-0.056** | **< .001** | **-0.026** | **0.004** | **-0.056** | **< .001** | **-0.026** | **0.004** | **-0.048** | **< .001** |
| Group | 0.025 | 0.044 | 0.052 | 0.575 | 0.024 | 0.044 | 0.052 | 0.579 | 0.030 | 0.044 | 0.064 | 0.496 | 0.063 | 0.048 | 0.119 | 0.188 |
| Neuroticism | **0.031** | **0.003** | **0.066** | **< .001** | **0.031** | **0.003** | **0.065** | **< .001** | **0.027** | **0.004** | **0.057** | **< .001** |  |  |  |  |
| Group*Neuroticism | 0.003 | 0.005 | 0.006 | 0.559 | 0.004 | 0.005 | 0.009 | 0.390 | 0.004 | 0.005 | 0.008 | 0.454 |  |  |  |  |
| Time*Neuroticism | **-0.001** | **0.000** | **-0.003** | **< .001** | **-0.001** | **0.000** | **-0.003** | **< .001** |  |  |  |  |  |  |  |  |
| Time*Group*Neuroticism | 0.000 | 0.001 | 0.001 | 0.478 |  |  |  |  |  |  |  |  |  |  |  |  |
| Time*Group | **0.023** | **0.004** | **0.0489** | **< .001** | **0.023** | **0.005** | **0.049** | **< .001** | **0.022** | **0.005** | **0.0461** | **< .001** | **0.022** | **0.005** | **0.041** | **< .001** |
| Gender | **0.166** | **0.046** | **0.351** | **< .001** | **0.165** | **0.046** | **0.350** | **< .001** | **0.169** | **0.046** | **0.356** | **< .001** | **0.221** | **0.050** | **0.416** | **< .001** |
| Education | -0.016 | 0.012 | -0.033 | 0.195 | -0.016 | 0.012 | -0.033 | 0.197 | -0.016 | 0.012 | -0.035 | 0.179 | -0.025 | 0.013 | -0.048 | 0.057 |
| Income | -0.019 | 0.013 | -0.040 | 0.159 | -0.019 | 0.013 | -0.040 | 0.158 | -0.018 | 0.013 | -0.039 | 0.175 | **-0.052** | **0.014** | **-0.098** | **< .001** |
| Stress |  |  |  |  |  |  |  |  |  |  |  |  |  |  |  |  |
| (Intercept) | **17.590** | **1.373** | **4.553** | **< .001** | **17.610** | **1.372** | **4.550** | **< .001** | **17.56** | **1.37** | **4.550** | **< .001** | **19.2** | **1.56** | **3.595** | **< .001** |
| Time | **-0.577** | **0.137** | **-0.149** | **< .001** | **-0.576** | **0.137** | **-0.149** | **< .001** | **-0.574** | **0.136** | **-0.149** | **< .001** | **-0.562** | **0.133** | **-0.105** | **< .001** |
| Group | -0.111 | 0.425 | -0.029 | 0.793 | -0.117 | 0.425 | -0.030 | 0.784 | -0.077 | 0.425 | -0.020 | 0.855 | 0.423 | 0.512 | 0.079 | 0.408 |
| Neuroticism | **0.462** | **0.035** | **0.120** | **< .001** | **0.446** | **0.034** | **0.115** | **< .001** | **0.422** | **0.032** | **0.109** | **< .001** |  |  |  |  |
| Group*Neuroticism | 0.015 | 0.049 | 0.004 | 0.762 | 0.046 | 0.044 | 0.012 | 0.292 | 0.042 | 0.044 | 0.011 | 0.333 |  |  |  |  |
| Time*Neuroticism | **-0.013** | **0.005** | **-0.003** | **0.006** | **-0.008** | **0.003** | **-0.002** | **0.013** |  |  |  |  |  |  |  |  |
| Time*Group*Neuroticism | 0.009 | 0.006 | 0.002 | 0.137 |  |  |  |  |  |  |  |  |  |  |  |  |
| Time*Group | **0.450** | **0.054** | **0.116** | **< .001** | **0.453** | **0.054** | **0.117** | **< .001** | **0.443** | **0.054** | **0.115** | **< .001** | **0.437** | **0.055** | **0.082** | **< .001** |
| Gender | 0.466 | 0.419 | 0.121 | 0.266 | 0.453 | 0.419 | 0.117 | 0.280 | 0.473 | 0.419 | 0.123 | 0.259 | **1.188** | **0.524** | **0.222** | **0.023** |
| Education | 0.0148 | 0.111 | 0.004 | 0.894 | 0.0156 | 0.111 | 0.004 | 0.889 | 0.013 | 0.111 | 0.003 | 0.907 | -0.120 | 0.140 | -0.023 | 0.389 |
| Income | 0.022 | 0.123 | 0.006 | 0.856 | 0.021 | 0.123 | 0.005 | 0.867 | 0.026 | 0.123 | 0.007 | 0.834 | **-0.498** | **0.151** | **-0.093** | **0.001** |

*Note.*

^a^ Model 4 assessed for a group by personality by time interaction

^b^ Model 3 assessed for a personality by time interaction

^c^ Model 2 assessed for the main effect of personality

^d^ Model 1 assessed assumes no effect of personality

Table 4

*Results of Linear Mixed Effect Modeling to assess the associations between the Extraversion personality dimensions and anxiety, depression, somatization symptoms and stress scores adjusted for gender, educational level, and income*

|  | Model 4^a^ | | | | Model 3^b^ | | | | Model 2^c^ | | | | Model 1^d^ | | | |
| --- | --- | --- | --- | --- | --- | --- | --- | --- | --- | --- | --- | --- | --- | --- | --- | --- |
| Variable | Estimate | SE | Cohen’s d | *p* | Estimate | SE | Cohen’s d | *p* | Estimate | SE | Cohen’s d | *p* | Estimate | SE | Cohen’s d | *p* |
| Anxiety | | | | | | | | | | | | | | | | |
| (Intercept) | **0.810** | **0.138** | **1.529** | **< .001** | **0.810** | **0.138** | **1.529** | **< .001** | **0.809** | **0.138** | **1.528** | **< .001** | **0.926** | **0.140** | **1.687** | **< .001** |
| Time | **-0.040** | **0.008** | **-0.076** | **< .001** | **-0.040** | **0.009** | **-0.078** | **< .001** | **-0.040** | **0.008** | **-0.076** | **< .001** | **-0.040** | **0.008** | **-0.073** | **< .001** |
| Group | 0.033 | 0.050 | 0.063 | 0.505 | 0.033 | 0.05 | 0.063 | 0.505 | 0.034 | 0.050 | 0.064 | 0.497 | 0.042 | 0.051 | 0.077 | 0.417 |
| Extraversion | **-0.017** | **0.005** | **-0.032** | **< .001** | **-0.017** | **0.005** | **-0.0320** | **< .001** | **-0.015** | **0.004** | **-0.029** | **0.001** |  |  |  |  |
| Group*Extraversion | -0.010 | 0.007 | -0.018 | 0.165 | -0.010 | 0.006 | -0.019 | 0.127 | -0.010 | 0.006 | -0.019 | 0.133 |  |  |  |  |
| Time*Extraversion | 0.001 | 0.001 | 0.001 | 0.275 | 0.001 | 0.000 | 0.001 | 0.169 |  |  |  |  |  |  |  |  |
| Time*Group*Extraversion | -0.0001 | 0.001 | -0.000 | 0.889 |  |  |  |  |  |  |  |  |  |  |  |  |
| Time*Group | **0.029** | **0.005** | **0.055** | **< .001** | **0.029** | **0.005** | **0.055** | **< .001** | **0.029** | **0.005** | **0.055** | **< .001** | **0.029** | **0.005** | **0.053** | **< .001** |
| Gender | 0.108 | 0.052 | 0.204 | 0.036 | 0.108 | 0.052 | 0.204 | 0.036 | **0.108** | **0.052** | **0.204** | **0.036** | 0.102 | 0.053 | 0.186 | 0.054 |
| Education | -0.007 | 0.014 | -0.013 | 0.63 | -0.007 | 0.014 | -0.013 | 0.630 | -0.007 | 0.014 | -0.0128 | 0.622 | -0.013 | 0.014 | -0.024 | 0.341 |
| Income | **-0.037** | **0.015** | **-0.069** | **0.015** | **-0.037** | **0.015** | **-0.069** | **0.015** | **-0.036** | **0.015** | **-0.069** | **0.016** | **-0.053** | **0.015** | **-0.096** | **0.001** |
| Depression |  |  |  |  |  |  |  |  |  |  |  |  |  |  |  |  |
| (Intercept) | **1.407** | **0.176** | **2.162** | **< .001** | **1.406** | **0.176** | **2.160** | **< .001** | **1.405** | **0.176** | **2.160** | **< .001** | **1.608** | **0.182** | **2.304** | **< .001** |
| Time | **-0.062** | **0.012** | **-0.095** | **< .001** | **-0.062** | **0.012** | **-0.095** | **< .001** | **-0.062** | **0.012** | **-0.095** | **< .001** | **-0.061** | **0.011** | **-0.088** | **< .001** |
| Group | 0.038 | 0.063 | 0.058 | 0.548 | 0.038 | 0.063 | 0.058 | 0.549 | 0.039 | 0.063 | 0.060 | 0.541 | 0.052 | 0.066 | 0.075 | 0.429 |
| Extraversion | **-0.027** | **0.006** | **-0.042** | **< .001** | **-0.028** | **0.006** | **-0.043** | **< .001** | **-0.026** | **0.006** | **-0.041** | **< .001** |  |  |  |  |
| Group*Extraversion | **-0.020** | **0.009** | **-0.031** | **0.021** | **-0.018** | **0.008** | **-0.027** | **0.029** | **-0.017** | **0.008** | **-0.027** | **0.030** |  |  |  |  |
| Time*Extraversion | 0.000 | 0.001 | 0.000 | 0.714 | 0.001 | 0.000 | 0.001 | 0.230 |  |  |  |  |  |  |  |  |
| Time*Group*Extraversion | 0.001 | 0.001 | 0.001 | 0.467 |  |  |  |  |  |  |  |  |  |  |  |  |
| Time*Group | **0.049** | **0.007** | **0.075** | **< .001** | **0.048** | **0.007** | **0.074** | **< .001** | **0.048** | **0.007** | **0.074** | **< .001** | **0.048** | **0.007** | **0.069** | **< .001** |
| Gender | **0.155** | **0.065** | **0.238** | **0.017** | **0.156** | **0.065** | **0.239** | **0.016** | **0.156** | **0.065** | **0.239** | **0.016** | **0.145** | **0.069** | **0.208** | **0.033** |
| Education | -0.023 | 0.017 | -0.036 | 0.176 | -0.023 | 0.017 | -0.036 | 0.175 | -0.024 | 0.017 | -0.036 | 0.171 | -0.035 | 0.018 | -0.050 | 0.053 |
| Income | **-0.038** | **0.019** | **-0.059** | **0.044** | **-0.038** | **0.019** | **-0.059** | **0.044** | **-0.038** | **0.019** | **-0.058** | **0.046** | **-0.066** | **0.020** | **-0.095** | **0.001** |
| Somatization |  |  |  |  |  |  |  |  |  |  |  |  |  |  |  |  |
| (Intercept) | **0.618** | **0.124** | **1.194** | **< .001** | **0.618** | **0.124** | **1.193** | **< .001** | **0.617** | **0.124** | **1.191** | **< .001** | **0.712** | **0.125** | **1.341** | **< .001** |
| Time | **-0.026** | **0.004** | **-0.050** | **< .001** | **-0.026** | **0.004** | **-0.050** | **< .001** | **-0.026** | **0.004** | **-0.050** | **< .001** | **-0.026** | **0.004** | **-0.048** | **< .001** |
| Group | 0.056 | 0.047 | 0.109 | 0.231 | 0.056 | 0.047 | 0.109 | 0.231 | 0.057 | 0.047 | 0.110 | 0.227 | 0.063 | 0.048 | 0.119 | 0.188 |
| Extraversion | **-0.013** | **0.004** | **-0.025** | **0.003** | **-0.013** | **0.004285** | **-0.025** | **0.002** | **-0.012** | **0.004** | **-0.023** | **0.005** |  |  |  |  |
| Group*Extraversion | -0.010 | 0.006 | -0.019 | 0.131 | -0.010 | 0.006 | -0.018 | 0.118 | -0.009 | 0.006 | -0.018 | 0.123 |  |  |  |  |
| Time*Extraversion | 0.000 | 0.000 | 0.001 | 0.347 | 0.000 | 0.000 | 0.002 | 0.172 |  |  |  |  |  |  |  |  |
| Time*Group*Extraversion | 0.000 | 0.001 | 0.000 | 0.945 |  |  |  |  |  |  |  |  |  |  |  |  |
| Time*Group | **0.022** | **0.005** | **0.042** | **< .001** | **0.022** | **0.005** | **0.042** | **< .001** | **0.022** | **0.005** | **0.042** | **< .001** | **0.022** | **0.005** | **0.041** | **< .001** |
| Gender | **0.225** | **0.049** | **0.434** | **< .001** | **0.225** | **0.049** | **0.434** | **< .001** | **0.225** | **0.049** | **0.434** | **< .001** | **0.221** | **0.050** | **0.416** | **< .001** |
| Education | -0.019 | 0.013 | -0.038 | 0.137 | -0.019 | 0.013 | -0.038 | 0.137 | -0.020 | 0.013 | -0.038 | 0.134 | -0.025 | 0.013 | -0.048 | 0.057 |
| Income | **-0.039** | **0.014** | **-0.076** | **0.006** | **-0.039** | **0.014** | **-0.076** | **0.006** | **-0.039** | **0.014** | **-0.075** | **0.007** | **-0.052** | **0.014** | **-0.098** | **< .001** |
| Stress |  |  |  |  |  |  |  |  |  |  |  |  |  |  |  |  |
| (Intercept) | **17.890** | **1.541** | **3.522** | **< .001** | **17.870** | **1.543** | **3.521** | **< .001** | **17.870** | **1.543** | **3.521** | **< .001** | **19.200** | **1.560** | **3.595** | **< .001** |
| Time | **-0.567** | **0.135** | **-0.112** | **< .001** | **-0.565** | **0.136** | **-0.111** | **< .001** | **-0.566** | **0.136** | **-0.112** | **< .001** | **-0.562** | **0.133** | **-0.105** | **< .001** |
| Group | 0.331 | 0.496 | 0.065 | 0.504 | 0.330 | 0.496 | 0.065 | 0.505 | 0.334 | 0.496 | 0.066 | 0.500 | 0.423 | 0.512 | 0.080 | 0.408 |
| Extraversion | **-0.162** | **0.046** | **-0.032** | **< .001** | **-0.183** | **0.045** | **-0.036** | **< .001** | **-0.173** | **0.043** | **-0.034** | **< .001** |  |  |  |  |
| Group*Extraversion | **-0.156** | **0.068** | **-0.031** | **0.021** | -0.112 | 0.063 | -0.022 | 0.074 | -0.111 | 0.063 | -0.022 | 0.078 |  |  |  |  |
| Time*Extraversion | -0.003 | 0.005 | -0.001 | 0.529 | 0.003 | 0.004 | 0.001 | 0.395 |  |  |  |  |  |  |  |  |
| Time*Group*Extraversion | 0.013 | 0.008 | 0.003 | 0.078 |  |  |  |  |  |  |  |  |  |  |  |  |
| Time*Group | **0.443** | **0.055** | **0.087** | **< .001** | **0.439** | **0.055** | **0.086** | **< .001** | **0.439** | **0.055** | **0.086** | **< .001** | **0.437** | **0.055** | **0.082** | **< .001** |
| Gender | **1.246** | **0.505** | **0.245** | **0.014** | **1.256** | **0.505** | **0.247** | **0.013** | **1.256** | **0.504** | **0.247** | **0.013** | **1.188** | **0.524** | **0.222** | **0.023** |
| Education | -0.042 | 0.135 | -0.008 | 0.757 | -0.041 | 0.135 | -0.008 | 0.758 | -0.042 | 0.135 | -0.008 | 0.756 | -0.120 | 0.140 | -0.023 | 0.389 |
| Income | **-0.318** | **0.148** | **-0.062** | **0.031** | **-0.318** | **0.148** | **-0.062** | **0.032** | **-0.316** | **0.148** | **-0.062** | **0.032** | **-0.498** | **0.151** | **-0.093** | **0.001** |

*Note.*

^a^ Model 4 assessed for a group by personality by time interaction

^b^ Model 3 assessed for a personality by time interaction

^c^ Model 2 assessed for the main effect of personality

^d^ Model 1 assessed assumes no effect of personality

Table 4

*Results of Linear Mixed Effect Modeling to assess the associations between the Openness personality dimensions and anxiety, depression, somatization symptoms and stress scores adjusted for gender, educational level, and income*

|  | Model 4^a^ | | | | Model 3^b^ | | | | Model 2^c^ | | | | Model 1^d^ | | | |
| --- | --- | --- | --- | --- | --- | --- | --- | --- | --- | --- | --- | --- | --- | --- | --- | --- |
| Variable | Estimate | SE | Cohen’s d | *p* | Estimate | SE | Cohen’s d | *p* | Estimate | SE | Cohen’s d | *p* | Estimate | SE | Cohen’s d | *p* |
| **Anxiety** | | | | | | | | | | | | | | | | |
| (Intercept) | **0.971** | **0.141** | **1.776** | **<.001** | **0.972** | **0.141** | **1.777** | **<.001** | **0.972** | **0.141** | **1.776** | **<.001** | **0.926** | **0.140** | **1.687** | **<.001** |
| Time | **-0.040** | **0.008** | **-0.073** | **<.001** | **-0.040** | **0.008** | **-0.073** | **<.001** | **-0.040** | **0.008** | **-0.073** | **<.001** | **-0.040** | **0.008** | **-0.073** | **<.001** |
| Group | 0.038 | 0.051 | 0.070 | 0.455 | 0.038 | 0.051 | 0.070 | 0.458 | 0.038 | 0.051 | 0.070 | 0.457 | 0.042 | 0.051 | 0.076 | 0.417 |
| Openness | 0.003 | 0.006 | 0.006 | 0.578 | 0.003 | 0.005 | 0.005 | 0.627 | 0.003 | 0.005 | 0.005 | 0.628 |  |  |  |  |
| Group*Openness | 0.008 | 0.008 | 0.015 | 0.313 | 0.009 | 0.008 | 0.017 | 0.229 | 0.009 | 0.008 | 0.017 | 0.228 |  |  |  |  |
| Time*Openness | -0.000 | 0.001 | -0.000 | 0.762 | -0.000 | 0.000 | -0.000 | 0.946 |  |  |  |  |  |  |  |  |
| Time*Group*Openness | 0.000 | 0.001 | 0.001 | 0.699 |  |  |  |  |  |  |  |  |  |  |  |  |
| Time*Group | **0.029** | **0.005** | **0.053** | **<.001** | **0.029** | **0.005** | **0.053** | **<.001** | **0.029** | **0.005** | **0.053** | **<.001** | **0.029** | **0.005** | **0.053** | **<.001** |
| Gender | 0.096 | 0.053 | 0.176 | 0.069 | 0.096 | 0.053 | 0.176 | 0.070 | 0.096 | 0.053 | 0.176 | 0.070 | 0.102 | 0.053 | 0.186 | 0.054 |
| Education | -0.019 | 0.014 | -0.036 | 0.178 | -0.019 | 0.014 | -0.036 | 0.177 | -0.019 | 0.014 | -0.036 | 0.177 | -0.013 | 0.014 | -0.024 | 0.341 |
| Income | **-0.052** | **0.015** | **-0.096** | **0.001** | **-0.052** | **0.015** | **-0.096** | **<.001** | **-0.052** | **0.015** | **-0.096** | **<.001** | **-0.053** | **0.015** | **-0.096** | **<.001** |
| **Depression** | | | | | | | | | | | | | | | | |
| (Intercept) | **1.57** | **0.184** | **2.248** | **<.001** | **1.57** | **0.184** | **2.248** | **<.001** | **1.571** | **0.185** | **2.25** | **<.001** | **1.608** | **0.182** | **2.304** | **<.001** |
| Time | **-0.061** | **0.011** | **-0.088** | **<.001** | **-0.061** | **0.011** | **-0.088** | **<.001** | **-0.061** | **0.011** | **-0.088** | **<.001** | **-0.061** | **0.011** | **-0.088** | **<.001** |
| Group | 0.056 | 0.066 | 0.081 | 0.397 | 0.056 | 0.066 | 0.080 | 0.398 | 0.055 | 0.066 | 0.079 | 0.405 | 0.052 | 0.066 | 0.075 | 0.429 |
| Openness | -0.010 | 0.007 | -0.014 | 0.178 | -0.010 | 0.007 | -0.014 | 0.155 | -0.008 | 0.007 | -0.012 | 0.213 |  |  |  |  |
| Group*Openness | 0.004 | 0.011 | 0.005 | 0.738 | 0.004 | 0.010 | 0.006 | 0.67 | 0.004 | 0.010 | 0.006 | 0.673 |  |  |  |  |
| Time*Openness | 0.000 | 0.001 | 0.001 | 0.604 | 0.001 | 0.001 | 0.001 | 0.396 |  |  |  |  |  |  |  |  |
| Time*Group*Openness | 0.000 | 0.001 | 0.000 | 0.846 |  |  |  |  |  |  |  |  |  |  |  |  |
| Time*Group | **0.048** | **0.007** | **0.068** | **<.001** | **0.048** | **0.007** | **0.068** | **<.001** | **0.048** | **0.007** | **0.069** | **<.001** | **0.048** | **0.007** | **0.069** | **<.001** |
| Gender | **0.152** | **0.068** | **0.217** | **0.026** | **0.152** | **0.068** | **0.217** | **0.027** | **0.151** | **0.068** | **0.217** | **0.027** | **0.145** | **0.068** | **0.208** | **0.033** |
| Education | -0.030 | 0.019 | -0.043 | 0.104 | -0.030 | 0.019 | -0.043 | 0.103 | -0.030 | 0.019 | -0.044 | 0.101 | -0.035 | 0.018 | -0.050 | 0.053 |
| Income | **-0.067** | **0.020** | **-0.095** | **<.001** | **-0.067** | **0.020** | **-0.095** | **<.001** | **-0.066** | **0.020** | **-0.095** | **<.001** | **-0.066** | **0.020** | **-0.095** | **<.001** |
| **Somatization** | | | | | | | | | | | | | | | | |
| (Intercept) | **0.710** | **0.127** | **1.335** | **<.001** | **0.71** | **0.127** | **1.335** | **<.001** | **0.71** | **0.127** | **1.335** | **<.001** | **0.712** | **0.125** | **1.341** | **<.001** |
| Time | **-0.026** | **0.004** | **-0.048** | **<.001** | **-0.026** | **0.004** | **-0.048** | **<.001** | **-0.026** | **0.004** | **-0.048** | **<.001** | **-0.026** | **0.004** | **-0.048** | **<.001** |
| Group | 0.063 | 0.048 | 0.119 | 0.189 | 0.063 | 0.048 | 0.119 | 0.188 | 0.063 | 0.048 | 0.119 | 0.188 | 0.063 | 0.048 | 0.119 | 0.188 |
| Openness | -0.000 | 0.005 | -0.000 | 0.997 | 0.000 | 0.005 | 0.000 | 0.984 | -0.000 | 0.005 | -0.000 | 0.989 |  |  |  |  |
| Group*Openness | -0.000 | 0.008 | -0.000 | 0.984 | -0.000 | 0.007 | -0.001 | 0.953 | -0.000 | 0.007 | -0.001 | 0.953 |  |  |  |  |
| Time*Openness | -0.000 | 0.001 | -0.000 | 0.973 | -0.000 | 0.000 | -0.000 | 0.882 |  |  |  |  |  |  |  |  |
| Time*Group*Openness | -0.000 | 0.001 | -0.000 | 0.903 |  |  |  |  |  |  |  |  |  |  |  |  |
| Time*Group | **0.022** | **0.005** | **0.041** | **<.001** | **0.022** | **0.005** | **0.0407** | **<.001** | **0.022** | **0.005** | **0.041** | **<.001** | **0.022** | **0.005** | **0.041** | **<.001** |
| Gender | **0.221** | **0.050** | **0.415** | **<.001** | **0.221** | **0.050** | **0.415** | **<.001** | **0.221** | **0.050** | **0.415** | **<.001** | **0.221** | **0.050** | **0.416** | **<.001** |
| Education | -0.025 | 0.014 | -0.047 | 0.066 | -0.025 | 0.014 | -0.047 | 0.066 | -0.025 | 0.014 | -0.047 | 0.067 | -0.025 | 0.013 | -0.048 | 0.057 |
| Income | **-0.052** | **0.014** | **-0.098** | **<.001** | **-0.052** | **0.014** | **-0.098** | **<.001** | **-0.052** | **0.014** | **-0.098** | **<.001** | **-0.052** | **0.014** | **-0.098** | **<.001** |
| **Stress** | | | | | | | | | | | | | | | | |
| (Intercept) | **19.02** | **1.583** | **3.56** | **<.001** | **19.02** | **1.584** | **3.561** | **<.001** | **19.01** | **1.581** | **3.559** | **<.001** | **19.2** | **1.56** | **3.595** | **<.001** |
| Time | **-0.564** | **0.134** | **-0.106** | **<.001** | **-0.563** | **0.134** | **-0.105** | **<.001** | **-0.562** | **0.133** | **-0.105** | **<.001** | **-0.562** | **0.133** | **-0.105** | **<.001** |
| Group | 0.429 | 0.513 | 0.080 | 0.403 | 0.425 | 0.513 | 0.080 | 0.407 | 0.438 | 0.513 | 0.082 | 0.393 | 0.423 | 0.512 | 0.079 | 0.408 |
| Openness | -0.043 | 0.055 | -0.0084 | 0.435 | -0.049 | 0.054 | -0.009 | 0.357 | -0.071 | 0.052 | -0.013 | 0.167 |  |  |  |  |
| Group*Openness | 0.060 | 0.082 | 0.011 | 0.467 | 0.075 | 0.077 | 0.014 | 0.330 | 0.075 | 0.077 | 0.014 | 0.328 |  |  |  |  |
| Time*Openness | -0.009 | 0.006 | -0.002 | 0.137 | -0.007 | 0.005 | -0.001 | 0.130 |  |  |  |  |  |  |  |  |
| Time*Group*Openness | 0.005 | 0.009 | 0.001 | 0.603 |  |  |  |  |  |  |  |  |  |  |  |  |
| Time*Group | **0.442** | **0.055** | **0.083** | **<.001** | **0.444** | **0.055** | **0.083** | **<.001** | **0.438** | **0.055** | **0.082** | **<.001** | **0.437** | **0.055** | **0.082** | **<.001** |
| Gender | **1.222** | **0.525** | **0.229** | **0.020** | **1.221** | **0.525** | **0.229** | **0.020** | **1.224** | **0.525** | **0.229** | **0.020** | **1.188** | **0.524** | **0.222** | **0.023** |
| Education | -0.100 | 0.143 | -0.019 | 0.484 | -0.100 | 0.143 | -0.019 | 0.483 | -0.098 | 0.143 | -0.018 | 0.493 | -0.120 | 0.140 | -0.023 | 0.389 |
| Income | **-0.500** | **0.151** | **-0.094** | **<.001** | **-0.5** | **0.151** | **-0.094** | **<.001** | **-0.501** | **0.151** | **-0.094** | **<.001** | **-0.498** | **0.151** | **-0.093** | **<.001** |

*Note.*

^a^ Model 4 assessed for a group by personality by time interaction

^b^ Model 3 assessed for a personality by time interaction

^c^ Model 2 assessed for the main effect of personality

^d^ Model 1 assessed assumes no effect of personality

Table 4

*Results of Linear Mixed Effect Modeling to assess the associations between the agreeableness personality dimensions and anxiety, depression, somatization symptoms and stress scores adjusted for gender, educational level, and income*

|  | Model 4^a^ | | | | Model 3^b^ | | | | Model 2^c^ | | | | Model 1^d^ | | | |
| --- | --- | --- | --- | --- | --- | --- | --- | --- | --- | --- | --- | --- | --- | --- | --- | --- |
| Variable | Estimate | SE | Cohen’s d | *p* | Estimate | SE | Cohen’s d | *p* | Estimate | SE | Cohen’s d | *p* | Estimate | SE | Cohen’s d | *p* |
| Anxiety | | | | | | | | | | | | | | | | |
| (Intercept) | **0.929** | **0.140** | **1.694** | **< .001** | **0.928** | **0.140** | **1.692** | **< .001** | **0.927** | **0.140** | **1.691** | **< .001** | **0.926** | **0.140** | **1.687** | **< .001** |
| Time | **-0.040** | **0.008** | **-0.072** | **< .001** | **-0.040** | **0.008** | **-0.072** | **< .001** | **-0.040** | **0.008** | **-0.073** | **< .001** | **-0.040** | **0.008** | **-0.073** | **< .001** |
| Group | 0.045 | 0.051 | 0.081 | 0.386 | 0.045 | 0.051 | 0.081 | 0.386 | 0.044 | 0.051 | 0.080 | 0.396 | 0.042 | 0.051 | 0.076 | 0.417 |
| Agreeableness | -0.005 | 0.006 | -0.009 | 0.405 | -0.004 | 0.006 | -0.007 | 0.491 | -0.006 | 0.006 | -0.012 | 0.262 |  |  |  |  |
| Group*Agreeableness | **0.018** | **0.009** | **0.033** | **0.049** | 0.016 | 0.009 | 0.029 | 0.069 | 0.016 | 0.009 | 0.029 | 0.072 |  |  |  |  |
| Time*Agreeableness | -0.000 | 0.001 | -0.001 | 0.459 | -0.001 | 0.000 | -0.001 | 0.093 |  |  |  |  |  |  |  |  |
| Time*Group*Agreeableness | -0.001 | 0.001 | -0.001 | 0.454 |  |  |  |  |  |  |  |  |  |  |  |  |
| Time*Group | **0.0291** | **0.005** | **0.053** | **< .001** | **0.029** | **0.005** | **0.053** | **< .001** | **0.029** | **0.005** | **0.053** | **< .001** | **0.029** | **0.005** | **0.053** | **< .001** |
| Gender | 0.100 | 0.054 | 0.182 | 0.066 | 0.010 | 0.054 | 0.182 | 0.067 | 0.010 | 0.054 | 0.182 | 0.066 | 0.102 | 0.053 | 0.186 | 0.054 |
| Education | -0.014 | 0.014 | -0.025 | 0.335 | -0.014 | 0.014 | -0.024 | 0.344 | -0.013 | 0.014 | -0.024 | 0.347 | -0.013 | 0.014 | -0.025 | 0.341 |
| Income | **-0.052** | **0.015** | **-0.095** | **0.001** | **-0.053** | **0.015** | **-0.096** | **0.001** | **-0.052** | **0.015** | **-0.095** | **0.001** | **-0.053** | **0.015** | **-0.096** | **0.001** |
| Depression |  |  |  |  |  |  |  |  |  |  |  |  |  |  |  |  |
| (Intercept) | **1.622** | **0.183** | **2.327** | **< .001** | **1.621** | **0.183** | **2.325** | **< .001** | **1.618** | **0.182** | **2.326** | **< .001** | **1.608** | **0.182** | **2.304** | **< .001** |
| Time | **-0.061** | **0.011** | **-0.088** | **< .001** | **-0.061** | **0.011** | **-0.087** | **< .001** | **-0.061** | **0.011** | **-0.088** | **< .001** | **-0.061** | **0.011** | **-0.088** | **< .001** |
| Group | 0.059 | 0.066 | 0.085 | 0.374 | 0.059 | 0.066 | 0.088 | 0.373 | 0.057 | 0.066 | 0.082 | 0.388 | 0.052 | 0.066 | 0.075 | 0.429 |
| Agreeableness | -0.006 | 0.008 | -0.008 | 0.471 | -0.003 | 0.008 | -0.005 | 0.650 | -0.008 | 0.007 | -0.011 | 0.288 |  |  |  |  |
| Group*Agreeableness | **0.031** | **0.012** | **0.0443** | **0.001** | **0.026** | **0.011** | **0.037** | **0.022** | **0.0254** | **0.011** | **0.037** | **0.024** |  |  |  |  |
| Time*Agreeableness | -0.001 | 0.001 | -0.001 | 0.380 | **-0.001** | **0.001** | **-0.002** | **0.020** |  |  |  |  |  |  |  |  |
| Time*Group*Agreeableness | -0.002 | 0.001 | -0.002 | 0.208 |  |  |  |  |  |  |  |  |  |  |  |  |
| Time*Group | **0.048** | **0.007** | **0.069** | **< .001** | **0.048** | **0.007** | **0.068** | **< .001** | **0.048** | **0.007** | **0.069** | **< .001** | **0.048** | **0.007** | **0.069** | **< .001** |
| Gender | 0.135 | 0.070 | 0.194 | 0.053 | 0.134 | 0.070 | 0.193 | 0.055 | 0.135 | 0.070 | 0.194 | **0.054** | **0.145** | **0.068** | **0.208** | **0.033** |
| Education | -0.035 | 0.018 | -0.051 | 0.051 | -0.035 | 0.018 | -0.050 | 0.055 | -0.035 | 0.018 | -0.050 | 0.056 | -0.035 | 0.018 | -0.050 | 0.053 |
| Income | **-0.065** | **0.020** | **-0.093** | **0.001** | **-0.065** | **0.020** | **-0.094** | **0.001** | **-0.065** | **0.020** | **-0.093** | **0.001** | **-0.066** | **0.020** | **-0.095** | **0.001** |
| Somatization |  |  |  |  |  |  |  |  |  |  |  |  |  |  |  |  |
| (Intercept) | **0.717** | **0.126** | **1.350** | **< .001** | **0.716** | **0.126** | **1.349** | **< .001** | **0.716** | **0.126** | **1.348** | **< .001** | **0.712** | **0.125** | **1.341** | **< .001** |
| Time | **-0.026** | **0.004** | **-0.048** | **< .001** | **-0.026** | **0.004** | **-0.048** | **< .001** | **-0.026** | **0.004** | **-0.048** | **< .001** | **-0.026** | **0.004** | **-0.048** | **< .001** |
| Group | 0.066 | 0.048 | 0.123 | 0.173 | 0.066 | 0.048 | 0.1234 | 0.173 | 0.065 | 0.048 | 0.123 | 0.175 | 0.063 | 0.048 | 0.119 | 0.188 |
| Agreeableness | -0.003 | 0.006 | -0.006 | 0.552 | -0.002 | 0.006 | -0.005 | 0.655 | -0.00 | 0.005 | -0.006 | 0.538 |  |  |  |  |
| Group*Agreeableness | 0.013 | 0.009 | 0.024 | 0.138 | 0.011 | 0.008 | 0.020 | 0.193 | 0.011 | 0.008 | 0.020 | 0.196 |  |  |  |  |
| Time*Agreeableness | < .001 | 0.001 | < .001 | 0.993 | -0.000 | 0.000 | -0.001 | 0.469 |  |  |  |  |  |  |  |  |
| Time*Group*Agreeableness | -0.001 | 0.001 | -0.001 | 0.417 |  |  |  |  |  |  |  |  |  |  |  |  |
| Time*Group | **0.022** | **0.005** | **0.041** | **< .001** | **0.021** | **0.005** | **0.040** | **< .001** | **0.021** | **0.005** | **0.040** | **< .001** | **0.022** | **0.005** | **0.041** | **< .001** |
| Gender | **0.217** | **0.051** | **0.408** | **< .001** | **0.216** | **0.051** | **0.407** | **< .001** | **0.216** | **0.051** | **0.408** | **< .001** | **0.221** | **0.050** | **0.416** | **< .001** |
| Education | -0.025 | 0.013 | -0.048 | 0.056 | -0.025 | 0.013 | -0.047 | 0.059 | -0.025 | 0.013 | -0.047 | 0.059 | -0.025 | 0.013 | -0.048 | 0.057 |
| Income | **-0.051** | **0.014** | **-0.097** | **0.001** | **-0.052** | **0.014** | **-0.097** | **< .001** | **-0.052** | **0.014** | **-0.097** | **< .001** | **-0.052** | **0.014** | **-0.098** | **< .001** |
| Stress |  |  |  |  |  |  |  |  |  |  |  |  |  |  |  |  |
| (Intercept) | **19.190** | **1.569** | **3.592** | **< .001** | **19.190** | **1.569** | **3.592** | **< .001** | **19.180** | **1.566** | **3.591** | **< .001** | **19.2** | **1.560** | **3.595** | **< .001** |
| Time | **-0.559** | **0.134** | **-0.105** | **< .001** | **-0.559** | **0.134** | **-0.105** | **< .001** | **-0.562** | **0.133** | **-0.105** | **< .001** | **-0.562** | **0.133** | **-0.105** | **< .001** |
| Group | 0.440 | 0.513 | 0.082 | 0.391 | 0.440 | 0.513 | 0.082 | 0.391 | 0.430 | 0.513 | 0.080 | 0.402 | 0.423 | 0.512 | 0.079 | 0.408 |
| Agreeableness | -0.040 | 0.061 | -0.008 | 0.509 | -0.042 | 0.059 | -0.008 | 0.480 | -0.066 | 0.057 | -0.012 | 0.247 |  |  |  |  |
| Group*Agreeableness | 0.127 | 0.093 | 0.024 | 0.170 | 0.131 | 0.087 | 0.024 | 0.132 | 0.128 | 0.087 | 0.024 | 0.141 |  |  |  |  |
| Time*Agreeableness | -0.009 | 0.007 | -0.002 | 0.201 | -0.001 | 0.005 | -0.002 | 0.103 |  |  |  |  |  |  |  |  |
| Time*Group*Agreeableness | 0.001 | 0.010 | 0.000 | 0.907 |  |  |  |  |  |  |  |  |  |  |  |  |
| Time*Group | **0.435** | **0.055** | **0.081** | **< .001** | **0.435** | **0.055** | **0.081** | **< .001** | **0.436** | **0.055** | **0.082** | **< .001** | **0.437** | **0.055** | **0.082** | **< .001** |
| Gender | **1.205** | **0.538** | **0.226** | **0.025** | **1.205** | **0.538** | **0.226** | **0.025** | **1.208** | **0.538** | **0.226** | **0.025** | **1.188** | **0.524** | **0.222** | **0.023** |
| Education | -0.120 | 0.140 | -0.022 | 0.390 | -0.121 | 0.140 | -0.023 | 0.388 | -0.120 | 0.140 | -0.022 | 0.390 | -0.120 | 0.140 | -0.023 | 0.389 |
| Income | **-0.501** | **0.151** | **-0.094** | **0.001** | **-0.501** | **0.151** | **-0.094** | **0.001** | **-0.498** | **0.151** | **-0.093** | **0.001** | **-0.498** | **0.151** | **-0.093** | **0.001** |

*Note.*

^a^ Model 4 assessed for a group by personality by time interaction

^b^ Model 3 assessed for a personality by time interaction

^c^ Model 2 assessed for the main effect of personality

^d^ Model 1 assessed assumes no effect of personality

Table 4

*Results of Linear Mixed Effect Modeling to assess the associations between the conscientiousness personality dimensions and anxiety, depression, somatization symptoms and stress scores adjusted for gender, educational level, and income*

|  | Model 4^a^ | | | | Model 3^b^ | | | | Model 2^c^ | | | | Model 1^d^ | | | |
| --- | --- | --- | --- | --- | --- | --- | --- | --- | --- | --- | --- | --- | --- | --- | --- | --- |
| Variable | Estimate | SE | Cohen’s d | *p* | Estimate | SE | Cohen’s d | *p* | Estimate | SE | Cohen’s d | *p* | Estimate | SE | Cohen’s d | *p* |
| Anxiety | | | | | | | | | | | | | | | | |
| (Intercept) | **0.829** | **0.139** | **1.546** | **< .001** | **0.829** | **0.139** | **1.545** | **< .001** | **0.827** | **0.139** | **1.543** | **< .001** | **0.926** | **0.140** | **1.687** | **< .001** |
| Time | **-0.040** | **0.008** | **-0.075** | **< .001** | **-0.040** | **0.008** | **-0.075** | **< .001** | **-0.040** | **0.008** | **-0.074** | **< .001** | **-0.040** | **0.008** | **-0.073** | **< .001** |
| Group | 0.023 | 0.051 | 0.042 | 0.655 | 0.023 | 0.051 | 0.043 | 0.652 | 0.024 | 0.051 | 0.045 | 0.638 | 0.042 | 0.051 | 0.076 | 0.417 |
| Conscientiousness | **-0.019** | **0.006** | **-0.036** | **0.001** | **-0.019** | **0.006** | **-0.035** | **0.001** | **-0.017** | **0.005** | **-0.032** | **0.002** |  |  |  |  |
| Group*Conscientiousness | -0.004 | 0.008 | -0.007 | 0.655 | -0.005 | 0.008 | -0.009 | 0.551 | -0.005 | 0.008 | -0.009 | 0.557 |  |  |  |  |
| Time*Conscientiousness | 0.001 | 0.001 | 0.001 | 0.295 | 0.000 | 0.000 | 0.001 | 0.269 |  |  |  |  |  |  |  |  |
| Time*Group*Conscientiousness | -0.000 | 0.001 | -0.001 | 0.724 |  |  |  |  |  |  |  |  |  |  |  |  |
| Time*Group | **0.030** | **0.005** | **0.055** | **< .001** | **0.030** | **0.005** | **0.055** | **< .001** | **0.029** | **0.005** | **0.055** | **< .001** | **0.0292** | **0.005** | **0.053** | **< .001** |
| Gender | **0.137** | **0.053** | **0.256** | **0.009** | **0.137** | **0.053** | **0.255** | **0.009** | **0.138** | **0.053** | **0.257** | **0.009** | 0.102 | 0.053 | 0.186 | 0.054 |
| Education | -0.015 | 0.014 | -0.028 | 0.286 | -0.015 | 0.014 | -0.027 | 0.290 | -0.0150 | 0.014 | -0.028 | 0.282 | -0.013 | 0.014 | -0.025 | 0.341 |
| Income | **-0.040** | **0.015** | **-0.074** | **0.010** | **-0.040** | **0.015** | **-0.074** | **0.010** | **-0.039** | **0.015** | **-0.073** | **0.001** | **-0.053** | **0.015** | **-0.096** | **0.001** |
| Depression |  |  |  |  |  |  |  |  |  |  |  |  |  |  |  |  |
| (Intercept) | **1.434** | **0.178** | **2.155** | **< .001** | **1.433** | **0.178** | **2.150** | **< .001** | **1.432** | **0.178** | **2.149** | **< .001** | **1.608** | **0.182** | **2.304** | **< .001** |
| Time | **-0.062** | **0.011** | **-0.093** | **< .001** | **-0.062** | **0.011** | **-0.092** | **< .001** | **-0.061** | **0.011** | **-0.092** | **< .001** | **-0.061** | **0.011** | **-0.088** | **< .001** |
| Group | 0.018 | 0.064 | 0.027 | 0.778 | 0.019 | 0.064 | 0.028 | 0.770 | 0.020 | 0.064 | 0.030 | 0.757 | 0.052 | 0.066 | 0.075 | 0.429 |
| Conscientiousness | **-0.033** | **0.007** | **-0.050** | **< .001** | **-0.032** | **0.007** | **-0.047** | **< .001** | **-0.030** | **0.007** | **-0.045** | **< .001** |  |  |  |  |
| Group*Conscientiousness | -0.006 | 0.011 | -0.010 | 0.549 | -0.011 | 0.010 | -0.016 | 0.290 | -0.011 | 0.010 | -0.016 | 0.294 |  |  |  |  |
| Time*Conscientiousness | 0.001 | 0.001 | 0.002 | 0.163 | 0.001 | 0.001 | 0.001 | 0.398 |  |  |  |  |  |  |  |  |
| Time*Group*Conscientiousness | -0.001 | 0.001 | -0.002 | 0.256 |  |  |  |  |  |  |  |  |  |  |  |  |
| Time*Group | **0.0491** | **0.007** | **0.074** | **< .001** | **0.049** | **0.007** | **0.073** | **< .001** | **0.048** | **0.007** | **0.072** | **< .001** | **0.048** | **0.007** | **0.069** | **< .001** |
| Gender | **0.210** | **0.066** | **0.316** | **0.002** | **0.209** | **0.066** | **0.313** | **0.002** | **0.210** | **0.066** | **0.315** | **0.002** | **0.145** | **0.068** | **0.208** | **0.033** |
| Education | **-0.038** | **0.018** | **-0.057** | **0.030** | **-0.038** | **0.018** | **-0.056** | **0.032** | **-0.038** | **0.018** | **-0.057** | **0.031** | -0.035 | 0.018 | -0.050 | 0.053 |
| Income | **-0.042** | **0.019** | **-0.064** | **0.028** | **-0.042** | **0.020** | **-0.064** | **0.028** | **-0.042** | **0.019** | **-0.063** | **0.028** | **-0.066** | **0.020** | **-0.095** | **0.001** |
| Somatization |  |  |  |  |  |  |  |  |  |  |  |  |  |  |  |  |
| (Intercept) | **0.620** | **0.124** | **1.194** | **< .001** | **0.619** | **0.124** | **1.192** | **< .001** | **0.617** | **0.124** | **1.189** | **< .001** | **0.712** | **0.125** | **1.341** | **< .001** |
| Time | **-0.026** | **0.004** | **-0.051** | **< .001** | **-0.026** | **0.004** | **-0.050** | **< .001** | **-0.026** | **0.004** | **-0.049** | **< .001** | **-0.026** | **0.004** | **-0.048** | **< .001** |
| Group | 0.045 | 0.047 | 0.086 | 0.344 | 0.045 | 0.047 | 0.087 | 0.340 | 0.046 | 0.047 | 0.089 | 0.326 | 0.063 | 0.048 | 0.119 | 0.188 |
| Conscientiousness | **-0.016** | **0.005** | **-0.032** | **0.002** | **-0.015** | **0.005** | **-0.029** | **0.004** | **-0.013** | **0.005** | **-0.026** | **0.009** |  |  |  |  |
| Group*Conscientiousness | -0.008 | 0.008 | -0.015 | 0.314 | -0.010 | 0.007 | -0.020 | 0.169 | -0.010 | 0.007 | -0.020 | 0.173 |  |  |  |  |
| Time*Conscientiousness | 0.001 | 0.001 | 0.002 | 0.058 | 0.001 | 0.000 | 0.001 | 0.104 |  |  |  |  |  |  |  |  |
| Time*Group*Conscientiousness | -0.001 | 0.001 | -0.002 | 0.301 |  |  |  |  |  |  |  |  |  |  |  |  |
| Time*Group | **0.022** | **0.005** | **0.043** | **< .001** | **0.022** | **0.005** | **0.043** | **< .001** | **0.022** | **0.005** | **0.042** | **< .001** | **0.022** | **0.005** | **0.041** | **< .001** |
| Gender | **0.253** | **0.050** | **0.487** | **< .001** | **0.252** | **0.050** | **0.485** | **< .001** | **0.253** | **0.050** | **0.487** | **< .001** | **0.221** | **0.050** | **0.416** | **< .001** |
| Education | **-0.026** | **0.013** | **-0.050** | **0.046** | **-0.026** | **0.013** | **-0.050** | **0.048** | **-0.026** | **0.013** | **-0.050** | **0.046** | -0.025 | 0.013 | -0.048 | 0.057 |
| Income | **-0.040** | **0.014** | **-0.077** | **0.006** | **-0.040** | **0.014** | **-0.077** | **0.006** | **-0.040** | **0.014** | **-0.076** | **0.006** | **-0.052** | **0.014** | **-0.098** | **< .001** |
| Stress |  |  |  |  |  |  |  |  |  |  |  |  |  |  |  |  |
| (Intercept) | **17.510** | **1.524** | **3.513** | **< .001** | **17.510** | **1.524** | **3.510** | **< .001** | **17.510** | **1.523** | **3.512** | **< .001** | **19.200** | **1.560** | **3.595** | **< .001** |
| Time | **-0.560** | **0.134** | **-0.112** | **< .001** | **-0.559** | **0.134** | **-0.112** | **< .001** | **-0.561** | **0.134** | **-0.112** | **< .001** | **-0.562** | **0.133** | **-0.105** | **< .001** |
| Group | 0.131 | 0.491 | 0.026 | 0.790 | 0.133 | 0.491 | 0.027 | 0.787 | 0.128 | 0.491 | 0.026 | 0.794 | 0.423 | 0.512 | 0.080 | 0.408 |
| Conscientiousness | **-0.279** | **0.056** | **-0.056** | **< .001** | **-0.274** | **0.054** | **-0.055** | **< .001** | **-0.280** | **0.052** | **-0.056** | **< .001** |  |  |  |  |
| Group*Conscientiousness | -0.083 | 0.081 | -0.017 | 0.308 | -0.094 | 0.076 | -0.019 | 0.214 | -0.095 | 0.076 | -0.019 | 0.212 |  |  |  |  |
| Time*Conscientiousness | -0.000 | 0.006 | -0.000 | 0.954 | -0.002 | 0.005 | -0.000 | 0.660 |  |  |  |  |  |  |  |  |
| Time*Group*Conscientiousness | -0.004 | 0.009 | -0.001 | 0.707 |  |  |  |  |  |  |  |  |  |  |  |  |
| Time*Group | **0.439** | **0.055** | **0.088** | **< .001** | **0.437** | **0.055** | **0.088** | **< .001** | **0.439** | **0.055** | **0.088** | **< .001** | **0.437** | **0.055** | **0.082** | **< .001** |
| Gender | **1.799** | **0.503** | **0.361** | **< .001** | **1.796** | **0.503** | **0.360** | **< .001** | **1.793** | **0.503** | **0.360** | **< .001** | **1.188** | **0.524** | **0.222** | **0.023** |
| Education | -0.144 | 0.133 | -0.029 | 0.277 | -0.143 | 0.133 | -0.029 | 0.280 | -0.143 | 0.133 | -0.029 | 0.282 | -0.120 | 0.140 | -0.023 | 0.389 |
| Income | -0.273 | 0.146 | -0.055 | 0.061 | -0.273 | 0.146 | -0.055 | 0.061 | -0.274 | 0.146 | -0.055 | 0.060 | **-0.498** | **0.151** | **-0.093** | **0.001** |

*Note.*

^a^ Model 4 assessed for a group by personality by time interaction

^b^ Model 3 assessed for a personality by time interaction

^c^ Model 2 assessed for the main effect of personality

^d^ Model 1 assessed assumes no effect of personality

Table 2S. Correlations between participants’ random slope estimates for time, across all four outcomes

|  | Anxiety | Depression | Somatization | Stress |
| --- | --- | --- | --- | --- |
| Anxiety | -- |  |  |  |
| Depression | .728*** | -- |  |  |
| Somatization | .745*** | .573*** | -- |  |
| Stress | .578*** | .649*** | .438*** | -- |

Note. *** p <.001.

Unconditional random slope (for time) mixed effects models were specified for each outcome; the random slope coefficients for time were outputted for each participant and coefficients were correlated.

Figure S1. Anxiety and Depression in average scores by high and low personality dimension scores for intervention and control group, as estimated by mixed linear effect models (model 3)

Note. The figures reflect the results from the models in which all personality dimensions were entered simultaneously as predictors.

*Figure S2.* Depression In Average Scores Over Time By High And Low Agreeableness Scores, As Estimated By Mixed Linear Effect Models (Model 3)
